# FOXM1-Driven CKS1B Upregulation Promotes Pancreatic Cancer Progression and Therapeutic Resistance

**DOI:** 10.7150/ijbs.105289

**Published:** 2025-01-13

**Authors:** Liuxi Zhang, Fang Wei, Qihui Sun, Xinyan Huang, Qi Zou, Mengmeng Jiang, Yuling Su, Shu Li, Xiaojia Li, Keping Xie, Jie He

**Affiliations:** 1 Guangzhou First People's Hospital and The Second Affiliated Hospital, South China University of Technology School of Medicine, #1 Panfu Road, Guangzhou, Guangdong 510180, P.R. China.; 2 Center for Pancreatic Cancer Research, South China University of Technology College of Medicine, 382 Waihuan Road, Guangzhou, Guangdong 510006, P.R. China.

**Keywords:** Pancreatic ductal adenocarcinoma, CKS1B, FOXM1, cell cycle, chemoresistance, targeted therapy.

## Abstract

Background: Pancreatic ductal adenocarcinoma (PDAC) remains a highly lethal malignancy with limited treatment options. Investigating novel therapeutic targets and understanding mechanisms of chemoresistance are crucial for improving patient outcomes. This study investigated the role of CKS1B in PDAC carcinogenesis, stemness and chemoresistance, and explores the underlying mechanisms driving its upregulation. The findings may provide novel therapeutic insights and potential strategies for the treatment of PDAC.

**Methods:** CKS1B expression was analyzed in PDAC tissues and cell lines, its impact on cell proliferation, migration, apoptosis, stemness and chemosensitivity were evaluated by using *in vitro* and *in vivo* models, and its underlying mechanistic connection to transcription factor FOXM1 was explored by using molecular biology methods.

**Results:** CKS1B was significantly upregulated in PDAC tissues and correlated with poor patient survival. CKS1B promoted PDAC cell proliferation, migration, and inhibited apoptosis. Expression of CKS1B enhanced the stemness properties of pancreatic cancer. CKS1B knockdown sensitized PDAC cells to the treatment of gemcitabine and oxaliplatin. Mechanistically, CKS1B is transcriptionally regulated by FOXM1, establishing a novel FOXM1-CKS1B signaling axis that regulates carcinogenesis, proliferation, migration, stemness, apoptosis, and drug resistance in PDAC.

**Conclusions:** Our findings strongly suggest that CKS1B plays a critical role in PDAC progression, stemness and chemoresistance. Targeting the FOXM1-CKS1B axis represents a promising therapeutic strategy for PDAC patients.

## Introduction

Pancreatic ductal adenocarcinoma (PDAC), with a predicted five-year survival rate of only 9%, remains a devastating disease with high mortality^1^, ^2^. This dismal prognosis stems from late-stage diagnosis, often with systemic metastases, highlighting the crucial need for novel therapeutic approaches^2^. Developing novel therapeutic approaches requires a deeper understanding of the mechanisms underlying PDAC development and progression.

Cell cycle dysregulation and compromised differentiation are hallmarks of malignant tumors. Pharmacological agents targeting specific cell cycle components, have shown promise in cancer treatment^3^-^7^. CKS1B, a protein with high functional conservation, plays a crucial role in cell cycle regulation. It acts as a cofactor for p27 ubiquitination^8^, interacts with CDK subunits^9^, ^10^ and is involved in G1/S transition^11^-^13^, G2/M arrest^14^, ^15^, and apoptosis across various cancers^16^-^18^. CKS1B overexpression has been linked to the progression of numerous malignancies^12^, ^14^, ^19^-^21^, underscoring its potential oncogenic role.

Recent studies have also implicated CKS1B in chemoresistance. Identified as a ubiquitin-like protein system resistance gene^22^, CKS1B induces resistance to inhibitors of ubiquitin-like protein synthesis. In PDAC, CKS1B expression has been shown to be upregulated^23^ and correlated with immune infiltration^24^. However, the mechanisms of gemcitabine resistance in PDAC are diverse and complex, and whether CKS1B influences gemcitabine resistance and its mechanisms remains a significant research gap. Cancer stem cells (CSCs) possess an abundance of protective drug transport proteins, conferring inherent resistance to chemotherapy^25^. Additionally, the pancreatic cancer stem cells (PCSCs) exhibit self-renewal, differentiation capacity, and insensitivity to therapeutic drugs^26^. Elucidating the mechanisms that maintain stemness in PDAC could provide new insights for overcoming chemoresistance.

Forkhead box protein M1 (FOXM1), a key cell-cycle regulator^27^-^29^, is also known to be involved in PDAC progression^30^-^32^. Elevated FOXM1 expression correlates with poor prognosis and promotes drug resistance by augmenting DNA damage repair, mitigating reactive oxygen species (ROS), and modulating tumor stemness^30^, ^33^. Although its role in advanced PDAC and acquired chemoresistance is not fully elucidated, FOXM1 presents a promising therapeutic target.

This study examined the expression of CKS1B and the mechanistic connection between CKS1B and FOXM1 in PDAC pathogenesis, investigated the function of CKS1B and FOXM1 in therapeutic resistance, and identified potential therapeutic strategies for PDAC treatment by targeting FOXM1-CKS1B axis.

## Results

### CKS1B upregulation in PDAC associates with poor patient prognosis

**Figure 1:**
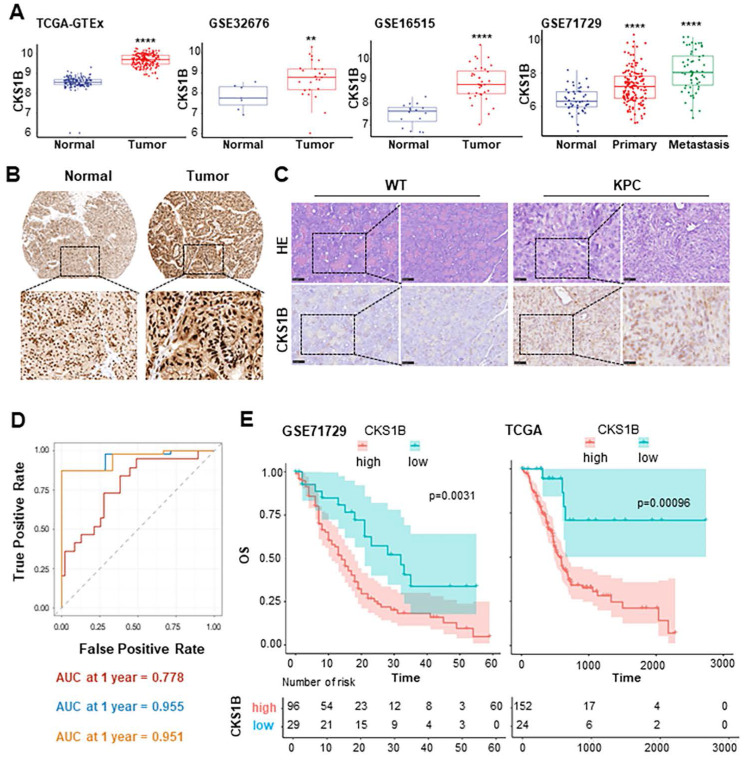
**CKS1B Upregulation in PDAC Associates with Poor Patient Prognosis. (A)** The expression of CKS1B in human pancreatic cancer analyzed using multiple datasets, including TCGA-GTEx, GSE32676, GSE16515, and GSE71729. *P*, 2.2e-16, 0.011, 7e-10 and 3.9e-14, respectively. **(B)** IHC staining of CKS1B protein expression in PDAC and normal pancreatic tissues from The Human Protein Atlas database. **(C)** H&E and IHC staining were used to assess CKS1B expression levels in pancreatic tissues from 33-week-old KPC mice, with wild-type (WT) mice serving as controls. **(D)** The AUC values for the ROC prognostic model were 0.778, 0.955 and 0.951 at 1, 3 and 5 years, respectively. **(E)** Kaplan-Meier OS curves of patients with PDAC according to CKS1B scores in TCGA and GSE71729 datasets.

Previous studies have reported CKS1B upregulation in certain types of tumors. To investigate its potential role of CKS1B and mechanism in PDAC, we first analyzed its expression in human PDAC using TCGA and GEO databases. We observed significantly higher CKS1B expression in PDAC tissues compared to that in normal pancreatic tissues (**Fig. 1A**). The finding was validated by immunohistochemistry (IHC) analysis from The Human Protein Atlas (https://www.proteinatlas.org/) and our own IHC staining on the mouse PDAC tissues from *LSL-Kras^G12D/+^; LSL-Trp53^R172H/+^; Pdx1-Cre* (KPC) (**Fig. 1B**, **1C**).

To assess the clinical significance of CKS1B expression in PDAC, we analyzed data from the TCGA PDAC cohort and found statistically significant associations between CKS1B expression and several patient characteristics, including history of radiotherapy, chronic pancreatitis history, age (<60 years), early-stage PDAC classification (Grade I), and T2 stage (**Supplementary Fig. 1**). Furthermore, receiver operating characteristic (ROC) curve analysis demonstrated that the area under the curve (AUC) values were 0.778, 0.955 and 0.951 at 1, 3, and 5 years, respectively, indicating that CKS1B expression effectively predicted patient prognosis (**Fig. 1D**). Notably, patients with high CKS1B expression had significantly shorter overall survival (OS) than that with low CKS1B expression (**Fig. 1E**). Therefore, CKS1B was upregulated in PDAC and negatively correlated with patient survival, highlighting its potential as a prognostic marker and therapeutic target in PDAC.

### CKS1B upregulation correlates with cell proliferation during PDAC development

To investigate the expression of CKS1B and its role in pancreatic carcinogenesis, we analyzed mouse single-cell sequencing data from the GSE141017 dataset. The analysis revealed that CKS1B was enriched in acinar-to-ductal metaplasia (ADM) lesion cell populations (**Supplementary Fig. 2A**). Further analysis of RNA-sequencing dataset GSE132326 demonstrated that CKS1B expression progressively increased during the transition from pancreatitis to PDAC, particularly in the context of *Kras* mutations and inflammation (**Supplementary Fig. 2B**). Therefore, CKS1B played a significant oncogenic role in PDAC progression.

To validate our findings, we established various animal models of pancreatitis and PDAC. We induced systemic inflammatory acute pancreatitis (AP) using L-arginine (L-ARG)^34^, ^35^ and cholestatic AP using Sodium Taurocholate^36^, ^37^ Histopathological examination revealed significant inflammation, including edema, hemorrhage, and necrosis, while Ki-67 positivity remained unchanged, consistent with CKS1B staining (**Supplementary Fig. 2C**).

**Figure 2:**
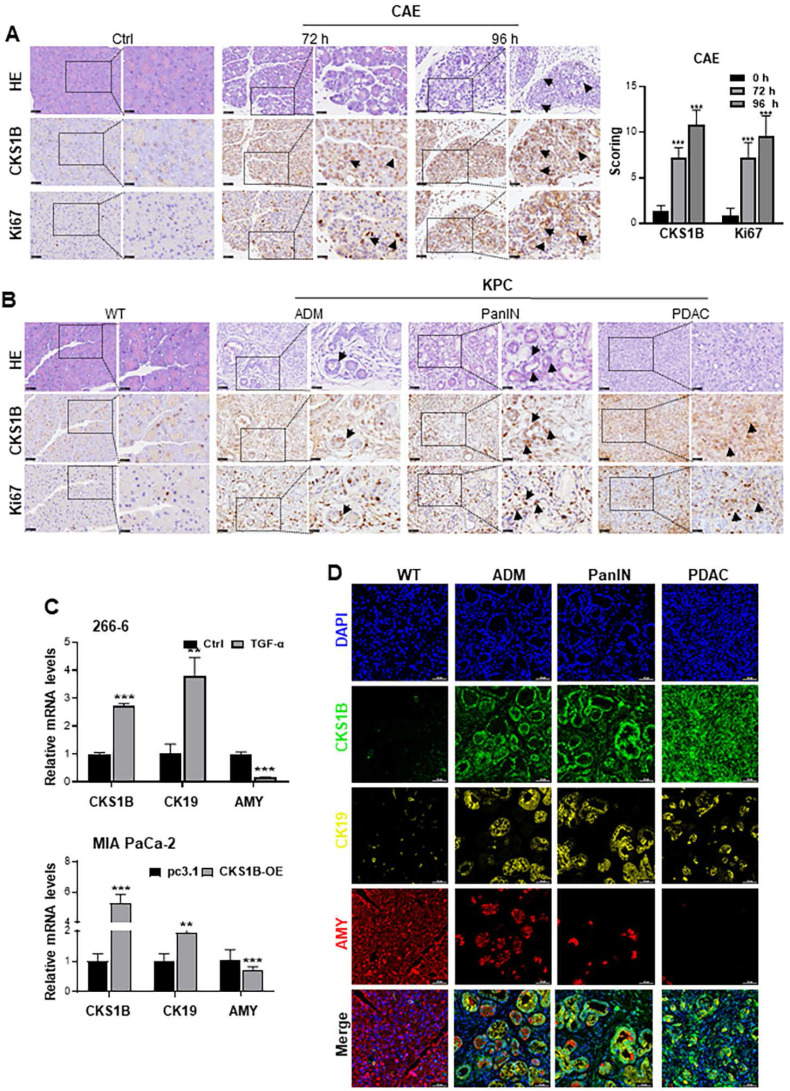
** CKS1B Upregulation Correlates with Cell Proliferation during PDAC Development. (A)** Expression of CKS1B and Ki67 was analyzed in ADM cells at 72 and 96 hours after CAE treatment by IHC. Untreated mice served as normal controls. **(B)** IHC demonstrated the upregulation of CKS1B and Ki67, as pancreatic lesions progressed from ADM and PanIN to PDAC in 33-week-old KPC mice, with WT mice serving as normal controls. Pancreatic lesions were confirmed by H&E staining. **(C)** RT-PCR analysis quantified CKS1B, CK19, and amylase “AMY” expression in 266-6 cells treated with TGF-α versus untreated controls, and in MIA PaCa-2 cells overexpressing CKS1B compared to pc3.1 vector controls. **(D)** Multiplex immunohistochemistry demonstrated the expression of CKS1B, CK19 and amylase, as pancreatic lesions evolved from ADM and PanIN to PDAC in 33-week-old KPC mice. WT mice served as normal controls. Scale bars: 50 μm.

We induced pancreatic ADM lesions using caerulein (CAE) treatment, pancreatic duct ligation (PDL), and genetically engineered *Kras-*mutant KC mice (*LSL-Kras^G12D/+^; Pdx1-Cre*). Histological analyses identified ADM lesions at 72 and 96 h following CAE treatment, with observed upregulation of both CKS1B and Ki67 within these lesions (**Fig. 2A**). Similar findings were noted in PDL-induced ADM at 72 h (**Supplementary Fig. 2D**). In the context of PDAC driven primarily by Kras mutations, increased expression of CKS1B and Ki67 was also detected in the KC-induced spontaneous ADM model compared to control areas (**Supplementary Fig. 2E**). Additionally, CKS1B mRNA levels were upregulated across these models (**Supplementary Fig. 2F**).

Mouse 266-6 cells were also treated with TGF-α to induce ADM. RT-PCR analysis showed that CKS1B expression was upregulated in the treated cells (**Fig. 2C**). Similarly, MIA PaCa-2 cells, which retains some acinar cell characteristics, also underwent ADM transformation following CKS1B overexpression (**Fig. 2C**). IHC analyses also revealed an increased CKS1B expression in ADM, pancreatic intraepithelial neoplasia (PanIN), and PDAC that spontaneously arose in KPC mice. This upregulation was significantly correlated with Ki67 staining (**Fig. 2B** and **Supplementary Fig. 2G**). Additionally, multiplex IHC (mIHC) validated these observations, indicating simultaneous upregulation of CKS1B and CK19, coupled with amylase downregulation during PDAC initiation and progression (**Fig. 2D** and **Supplementary Fig. 2H**). These findings collectively suggest a critical link between CKS1B expression and the development and progression of PDAC and further support the role of CKS1B in promoting pancreatic carcinogenesis.

### CKS1B overexpression enhances growth and migration of PDAC cells

Analysis of 12 pancreatic cancer cell lines revealed a generally elevated CKS1B expression compared to normal human pancreatic duct epithelial (HPDE) cells. Notably, BxPC-3 and CFPAC-1 cells displayed higher levels of CKS1B expression, whereas PL45 and MIA PaCa-2 cells exhibited lower levels (**Supplementary Fig. 3A**). Consequently, these four cell lines were chosen to manipulate CKS1B expression for further experiments (**Supplementary Fig. 3B**, **3C**).

**Figure 3:**
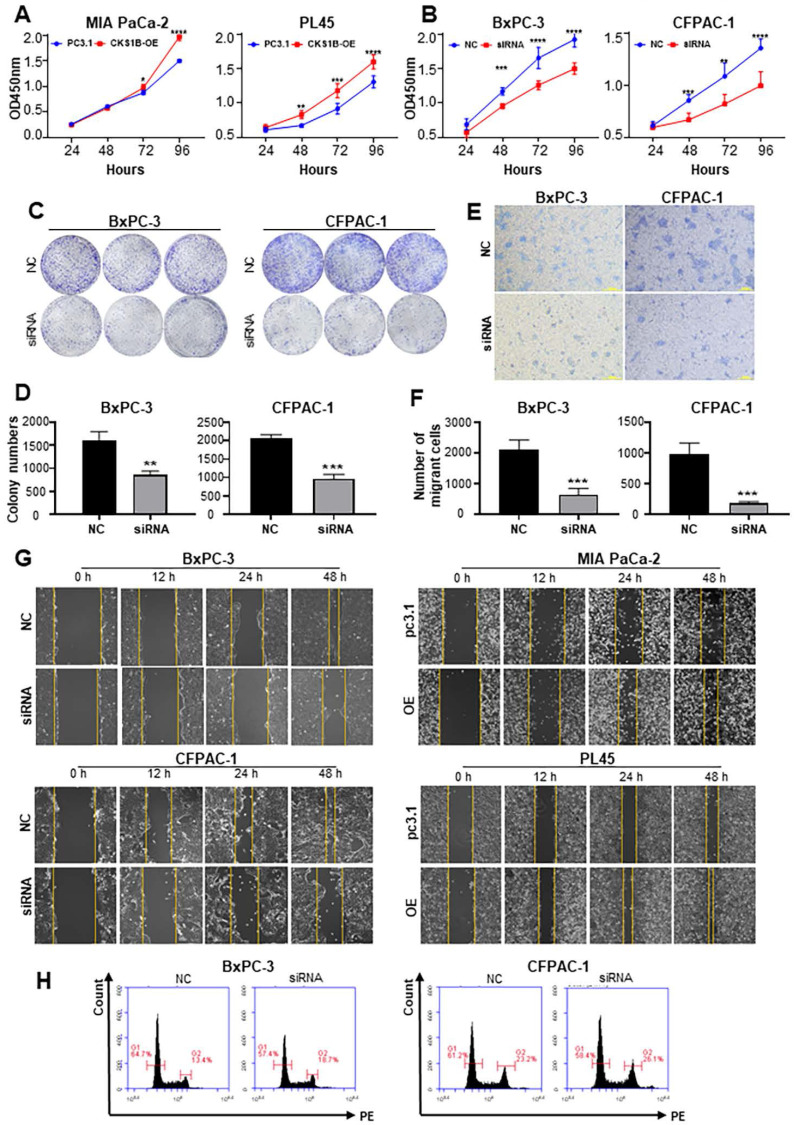
** CKS1B Overexpression Enhances Growth and Migration of PDAC Cells. (A)** CCK-8 assay revealed a significant increase in proliferation of PL45 and MIA PaCa-2 cells overexpressing CKS1B. **(B)**CCK-8 assay demonstrated a significant decrease in proliferation of BxPC-3 and CFPAC-1 cells following CKS1B knockdown. **(C-D)** Colony formation assays showed reduced proliferation in BxPC-3 and CFPAC-1 cell lines following CKS1B knockdown. **(E-F)** Migration ability of BxPC-3 and CFPAC-1 cell lines with CKS1B knockdown by transwell assays. Scale bars: 100 μm. **(G)** Wound healing assays in PDAC cells transfected with CKS1B overexpression or knockdown, with pc3.1 or NC as controls. Scale bars: 400 μm. **(H)** Flow cytometry analysis of the effect of CKS1B expression on the cell cycle.

CKS1B overexpression promoted cell growth in PL45 and MIA PaCa-2 cells (**Fig. 3A**), whereas CKS1B knockdown suppressed cell growth and colony formation in BxPC-3 and CFPAC-1 cells (**Fig. 3B-D**). CKS1B knockdown decreased migration ability in BxPC-3 and CFPAC-1 cells, while CKS1B overexpression enhanced migration in PL45 and MIA PaCa-2 cells (**Fig. 3E-G** and **Supplementary Fig. 3D**). Additionally, CKS1B knockdown significantly hindered the G2 to mitosis transition (**Fig. 3H** and **Supplementary Fig. 3D**). These findings suggest that CKS1B plays a critical role in promoting cell growth and migration in PDAC cells.

### CKS1B expression enhances the stemness properties of PDAC

**Figure 4:**
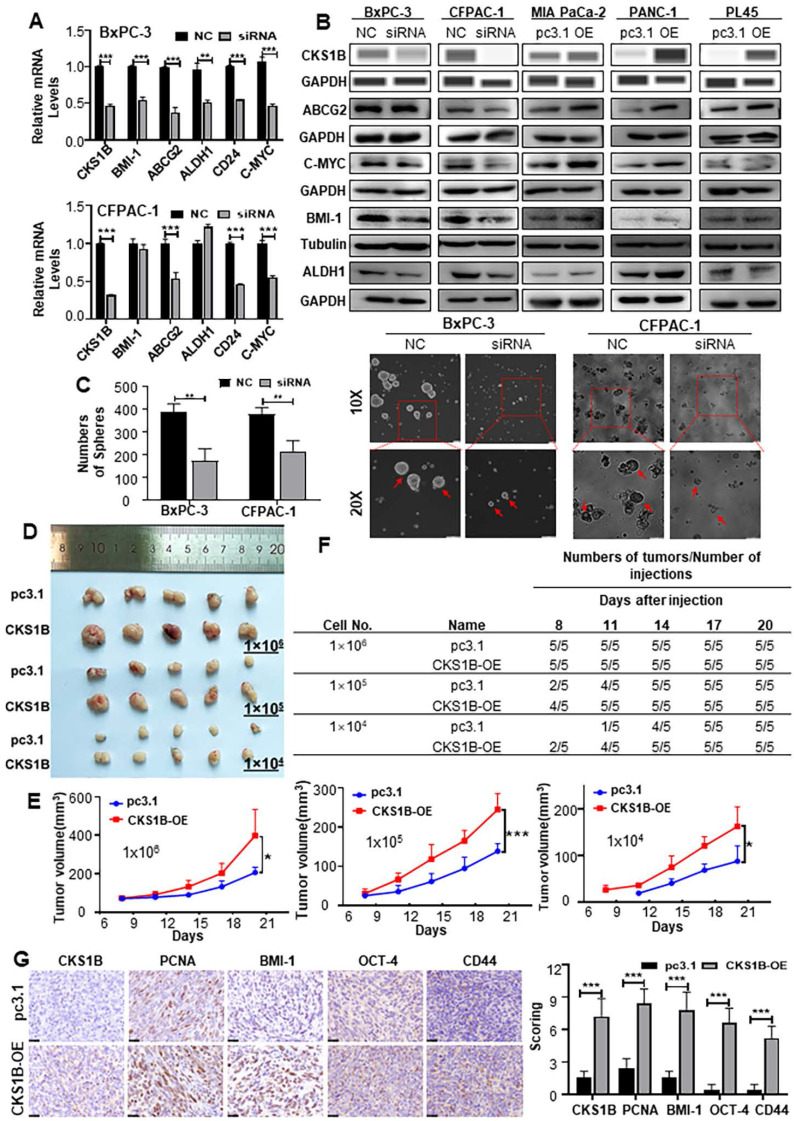
** CKS1B Expression Enhances the Stemness Properties of PDAC. (A)** RT-PCR analysis of cancer stem cell markers Bmi-1, ABCG2, ALDH1, CD24, and C-MYC in CKS1B knockdown PDAC cell lines, with NC as the control. **(B)** The protein levels of CKS1B, ABCG2, C-MYC, ALDH1, and Bmi-1 in CKS1B-overexpressing and CKS1B-knockdown PDAC cells were assessed using Western blot analysis. **(C)** Tumor sphere size and density were analyzed to assess the sphere-forming ability of PDAC cells with CKS1B knockdown or control (NC). Scale bars: 100 μm. **(D)** For the *in vivo* limiting dilution assay, CKS1B-overexpressing and pc3.1 control cells were subcutaneously injected into C57BL/6J mice. A representative image was shown. **(E)** Tumor growth curves in C57BL/6J mice injected with Panc02 cells, either overexpressing CKS1B or harboring the control vector (“pc3.1”), CKS1B-overexpressing tumors exhibited a significantly higher growth rate compared to the vector control group. Significant differences in tumor growth were observed at inoculation of 10⁶, 10⁵, and 10⁴ cells per mouse. **(F)** Tumors derived from CKS1B-overexpressing cells formed earlier and grew more rapidly compared to those from control cells. Tumor formation was monitored for 21 days post-injection. **(G)** The expression levels of CKS1B, PCNA, Bmi-1, OCT-4 and CD44 in CKS1B-overexpressing or control tumors were determined by IHC. Scale bars: 25 μm.

Many malignant behaviors of tumors are closely related to the stem cell-like properties of tumors^38^, ^39^. To investigate whether CKS1B could increase the stemness properties of PDAC, we analyzed human RNA-sequencing data from TCGA. We observed a robust correlation between CKS1B expression and PDAC stemness (**Supplementary Fig. 4A**). Further analysis of stemness signature proteins, including ABCG2, C-MYC, ALDH1, BMI-1 and CD24, which are commonly used for the detection of PCSCs^40^-^42^, revealed that the upregulation of CKS1B is positively correlated with the expression of these markers (**Fig. 4A** and **4B**).

Both CD44 and CD24 positive pancreatic cancer cells are considered PCSCs^43^. Immunocytochemical analysis showed that the ratio of CD24^+^/CD44^+^ cells was significantly affected by CKS1B (**Supplementary Fig. 4E**). Additionally, the proportion of CD133^+^ cells, also recognized as PCSCs, was notably affected by CKS1B expression (**Supplementary Fig. 4F**). The sphere formation assay, a classical method to measure the self-renewal property of CSCs *in vitro*^44^, demonstrated that overexpression and downregulation of CKS1B significantly influenced both the size and number of spheres formed by PDAC cells (**Fig. 4C**, **Supplementary Fig. 4B**, **4C**).

Consistent with the* in vitro* results, *in vivo* limiting dilution tumorigenicity assays showed that tumors overexpressing CKS1B grew at a significantly faster rate than control tumors. When the number of injected cells was reduced to 1×10^5^ or 1×10^4^, palpable tumors appeared earlier in the CKS1B-overexpressing group compared to the control group (**Fig. 4D**-**4F**). The tumors formed from CKS1B-overexpressing PDAC cells were larger than those formed from control cells (**Supplementary Fig. 4D**). Furthermore, expression levels of CKS1B, PCNA, BMI-1, OCT-4, and CD44 were higher in the CKS1B-overexpressing tumors compared to the control tumors (**Fig. 4G**). Collectively, our data demonstrate that overexpression of CKS1B significantly increases the frequency of CSCs in pancreatic cancer cells.

### CKS1B mediates drug resistance in PDAC cells by blocking gemcitabine and oxaliplatin-induced apoptosis

**Figure 5:**
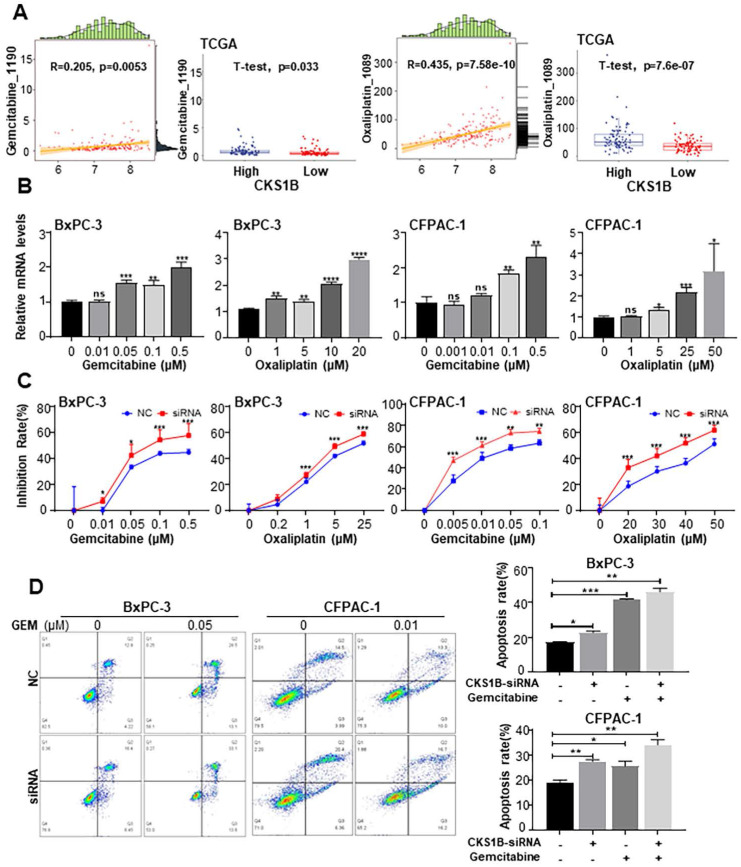
** CKS1B Mediates Drug Resistance in PDAC Cells by Blocking Gemcitabine and Oxaliplatin-Induced Apoptosis. (A)** Left: Correlation between CKS1B expression and responsiveness to Oxaliplatin and Gemcitabine, illustrated by a scatter plot. The horizontal axis showed CKS1B expression levels, and the vertical axis represented drug expression levels. Data points were annotated with correlation coefficients and P-values. Right: Boxplots depicting IC50 values for Oxaliplatin and Gemcitabine in CKS1B high-risk and low-risk groups, analyzed using the T-test. **(B)** RT-PCR analysis of CKS1B after 72 h of treatment with Gemcitabine and Oxaliplatin at varying concentrations. **(C)** Inhibition rate of BxPC-3 and CFPAC-1 cell lines with CKS1B knockdown following treatment with varying concentrations of gemcitabine or oxaliplatin at 72 h. **(D)** Flow cytometry analysis revealed differential apoptosis rates between NC and CKS1B knockdown groups of BxPC-3 and CFPAC-1 cells, with or without Gemcitabine treatment.

Previous studies have identified CKS1B as a potential resistance gene that enhances tumor cell drug resistance in multiple myeloma^45^ and lung cancer^46^. Additionally, CKS1B has been shown to selectively induce resistance to inhibitors of ubiquitin-like protein synthesis, but not to other antitumor drugs^22^. To investigate the association between CKS1B expression and chemotherapy resistance in PDAC, as well as to elucidate the underlying mechanisms, we downloaded the expression data of TCGA-PAAD from UCSC XENA based on the GDSC drug database, and then used as a test data for the “calcPhenotype” function of the “oncoPredict” package. We revealed strong correlations between CKS1B expression and sensitivity to both gemcitabine and oxaliplatin (**Supplementary Fig. 5A**-**5B**). Additionally, cells with elevated CKS1B expression exhibited significantly higher IC50 values for these drugs, indicating enhanced drug resistance (**Fig. 5A**).

To investigate the impact of CKS1B on drug sensitivity, BxPC-3 and CFPAC-1 cells were treated with increasing concentrations of gemcitabine and oxaliplatin. We observed that CKS1B expression increased in response to drug treatment (**Fig. 5B**). Notably, knockdown of CKS1B significantly enhanced the sensitivity of these cells to both drugs, resulting in increased growth inhibition (**Fig. 5C**).

Deregulated apoptotic signaling can lead to uncontrolled proliferation, resulting in tumor survival, therapeutic resistance and cancer recurrence^47^, ^48^. To determine whether CKS1B contributes to chemotherapy resistance by inhibiting apoptosis, flow cytometry analysis was conducted. The results revealed a significant increase in apoptotic cells following CKS1B knockdown, particularly after treatment with gemcitabine (**Fig. 5D**). Additionally, CFPAC-1 cells with high CKS1B expression exhibited a reduced percentage of apoptosis compared to HPAC cells with low expression (**Supplementary Fig. 5C**). Immunocytochemistry revealed elevated levels of cleaved caspase-3 and cleaved caspase-9 in the CKS1B-knockdown cells, indicating activation of the apoptotic pathway. Notably, this effect was most pronounced in cells co-treated with gemcitabine (**Supplementary Fig. 5D**). These findings indicate that CKS1B is crucial in mediating chemoresistance in pancreatic cancer through apoptosis inhibition. Consequently, targeting CKS1B may represent a promising strategy to overcome drug resistance and enhance treatment efficacy.

### CKS1B enhances proliferation, resistance, and stemness of PDAC *in vivo*

**Figure 6:**
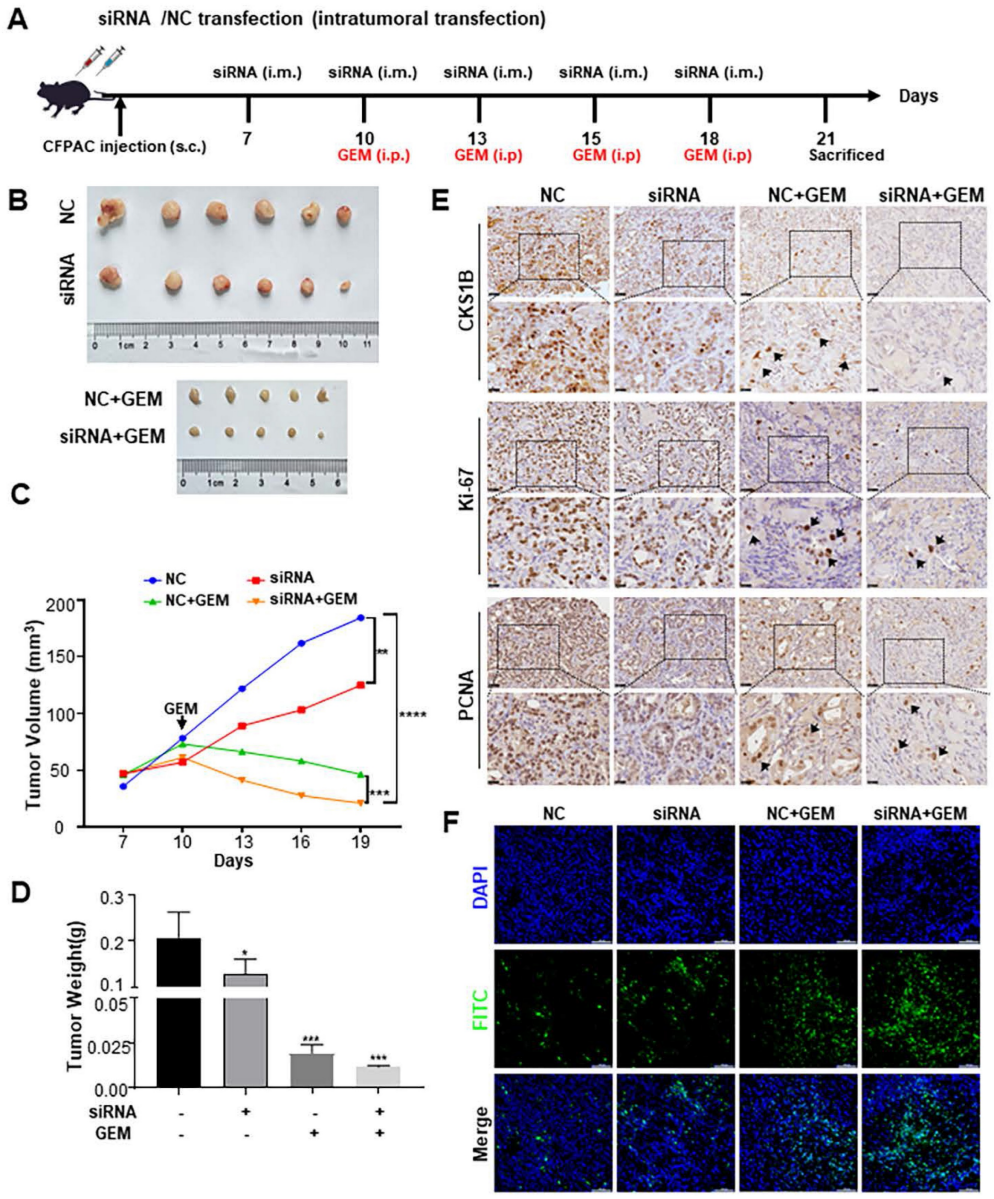
** CKS1B Enhances Proliferation, Resistance, and Stemness of PDAC *In Vivo.* (A)** Assessment of the impact of CKS1B on PDAC tumor growth *in vivo*. **(B)** CFPAC-1 cells were subcutaneously injected into NOD/SCID mice, and tumors were transfected with NC-siRNA or CKS1B-siRNA (n=6), followed by Gemcitabine treatment (n=5). A representative image was shown. **(C)** Tumor volume were assessed in mice after different treatments (n=6) with Gemcitabine (n =5). **(D)** Tumor weight were assessed in mice after different treatments (n=6) with Gemcitabine (n=5). **(E)** The expression levels of CKS1B, Ki-67, and PCNA in tumors were assessed by IHC. **(F)** Apoptosis rate in tumor tissues was analyzed using the TUNEL assay. Scale bars: 100 μm.

To assess the impact of CKS1B on the malignant phenotype of PDAC *in vivo*, nude mice were subcutaneously injected with CFPAC-1 cells and divided into four groups: negative control siRNA (“NC”), siRNA, NC+GEM, and siRNA+GEM. These groups received intratumoral CKS1B-siRNA injections and intraperitoneal gemcitabine injections (**Fig. 6A**). Knockdown of CKS1B significantly reduced tumor growth rate and overall tumor weight compared to the control groups (**Fig. 6B**-**6D**). Gemcitabine treatment further delayed tumor growth in both NC+GEM and siRNA+GEM groups, with the latter exhibiting the most pronounced regression (**Fig. 6B**-**6D**). No significant side effects were observed in mice treated with CKS1B-siRNA or the combination therapy (data not shown).

Histological analysis revealed a smaller necrotic area in siRNA and siRNA +GEM groups, indicating slower tumor growth (**Supplementary Fig. 6A**). Immunostaining for Ki67 and PCNA showed a decreased proliferation in tumors treated with siRNA and siRNA+GEM, while TUNEL assays demonstrated increased apoptosis in these groups compared to controls (**Fig. 6E**, **6F**, **Supplementary Fig. 6B**, **6C**). Immunocytochemical analysis showing elevated levels of cleaved caspase-3 and cleaved caspase-9 in the CKS1B-knockdown group compared to control, especially after gemcitabine treatment (**Supplementary Fig. 6D**).

To investigate whether CKS1B in PDAC modulates CSCs and contributes to chemotherapy resistance, IHC analysis of tumor samples was performed. CKS1B knockdown decreased the expression of BMI-1, OCT-4, CD44 and SOX2, particularly after gemcitabine treatment (**Supplementary Fig. 6E**). These findings collectively suggest that CKS1B inhibition suppresses tumor growth and enhances the efficacy of gemcitabine by promoting apoptosis, and CKS1B may facilitate chemotherapy resistance through its regulation of CSCs. This intricate link warrants further investigation to fully elucidate the therapeutic potential of targeting CKS1B in PDAC.

### CKS1B mediates FOXM1-regulated cell proliferation, migration and stemness

**Figure 7:**
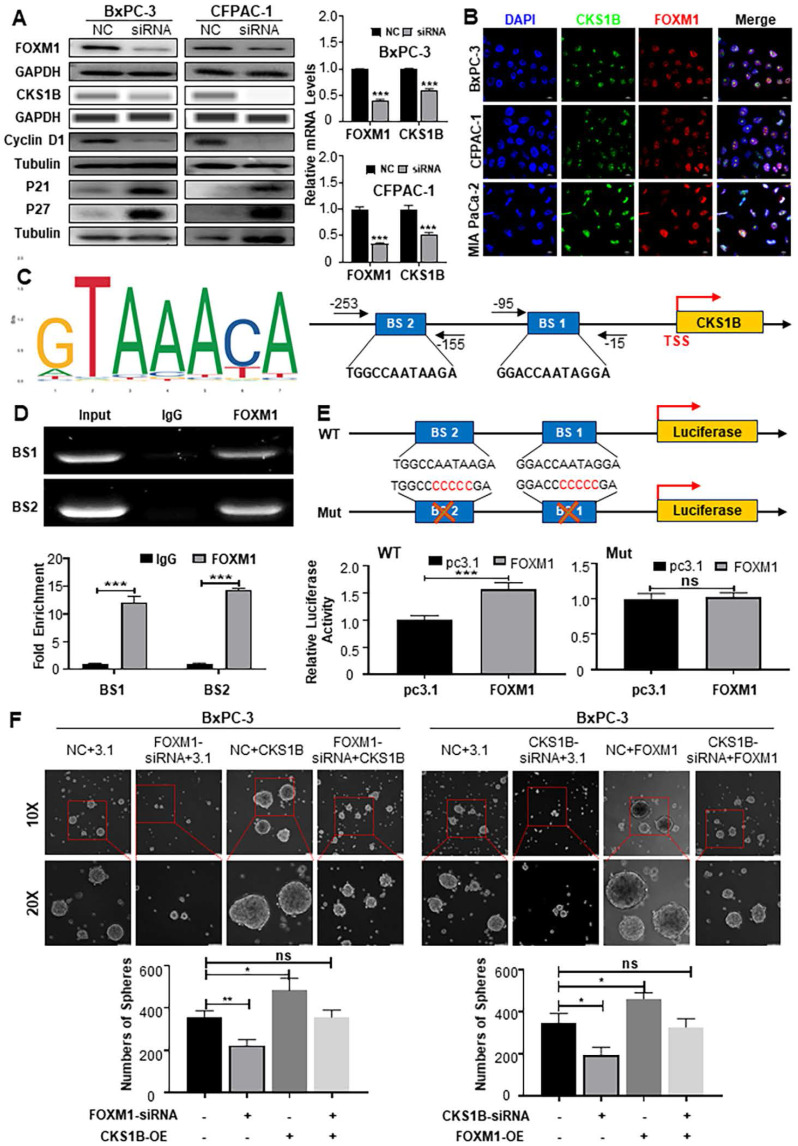
**FOXM1 Enhances the Malignant Phenotype of PDAC by Upregulating CKS1B Expression. (A)** Protein levels of FOXM1, CKS1B, cyclinD1 and P27 in PDAC cells transfected with FOXM1-siRNA, while their RNA expression was assessed using RT-PCR. **(B)** Co-localization of FOXM1 and CKS1B observed in human PDAC cells, Scale bars: 10 μm. **(C)** Schematic FoxM1 conserved binding site, predicted FoxM1 binding sites in CKS1B promoter by GTRD and JASPAR database, and primer pairs for ChIP. **(D)** ChIP assay to determine the binding of FOXM1 to the promoter of CKS1B. IgG was used as a negative control. Representative results were shown. **(E)** Luciferase reporter assay with elimination of FoxM1 binding sites by base mutation in the promoter of CKS1B. **(F)** Tumor sphere size and density indicated that CKS1B overexpression rescued the inhibition of tumor sphere formation capacity caused by FOXM1 knockdown, whereas CKS1B knockdown reduced the promotion of tumor sphere formation capacity induced by FOXM1 overexpression in PDAC cells.

To elucidate the mechanisms underlying CKS1B upregulation in PDAC and its association with malignancy, we leveraged the GEPIA platform to reveal a strong positive correlation between CKS1B and the transcription factor FOXM1 (http://gepia.cancer-pku.cn/index.html; **Supplementary Fig. 7A**). As expected, FOXM1 knockdown dramatically decreased both protein and mRNA levels of CKS1B in human PDAC cells, accompanied by a cell cycle blockade (**Fig. 7A**). Conversely, FOXM1 overexpression led to a substantial increase in CKS1B expression (**Supplementary Fig. 7B**, **7C**). Subcellular localization studies in human PDAC cells and tissues revealed nuclear expression of FOXM1 and CKS1B, suggesting their potential functional interaction (**Fig. 7B**, **Supplementary Fig. 7D**). This finding aligns with previous reports demonstrating FOXM1-mediated CKS1B regulation in osteosarcoma^49^.

To confirm CKS1B as a direct target of FOXM1, we identified potential FOXM1 binding sites within the CKS1B promoter using the JASPAR database (**Fig. 7C**). Chromatin immunoprecipitation (ChIP) assays confirmed the presence of two predicted binding sites, BS1 (-15 to -95) and BS2 (-155 to -253) (**Fig. 7D**). Further validation was provided by luciferase reporter assays using vectors with either the intact or mutated CKS1B promoter. In cells overexpressing FOXM1, the intact promoter luciferase activity was significantly enhanced, whereas the mutated promoter, lacking functional FOXM1 binding sites, showed no response (**Fig. 7E**). These results highlight the essential role of FOXM1-driven CKS1B transcriptional activation.

Given the established role of FOXM1 promoting PDAC progression, we next investigated whether CKS1B was involved in the PDAC malignant phenotype regulated by FOXM1. We overexpressed CKS1B in BxPC-3 and CFPAC-1 cells with either FOXM1 knockdown or control expression (**Supplementary Fig. 7E**, **7F**). Overexpression of CKS1B reversed the suppressed tumor growth (**Supplementary Fig. 7G**), migration (**Supplementary Fig. 7H**, **7I**) and CSCs self-renewal (**Fig. 7F**) observed in FOXM1-knockdown cells compared to their control counterparts. Conversely, knocking down CKS1B in BxPC-3 and CFPAC-1 cells overexpressing FOXM1 (**Supplementary Fig. 7J**, **7K**) demonstrated that CKS1B knockdown reversed the enhanced tumor growth (**Supplementary Fig. 7L**), migration (**Supplementary Fig. 7M**, **7N**), and CSCs self-renewal (**Fig. 7F**) associated with FOXM1 overexpression. Collectively, our data strongly indicate that FOXM1 upregulates CKS1B expression transcriptionally in PDAC cells and CKS1B is instrumental in mediating FOXM1-driven tumor proliferation, migration, and stemness, thereby promoting tumor progression.

## Discussion

PDAC remains a highly aggressive malignancy with limited therapeutic options. Gemcitabine is the mainstay of treatment, but chemoresistance is a major hurdle leading to poor clinical outcomes^1^, ^50^. Chemoresistance in PDAC is a complex phenomenon involving interactions between cancer cells, CSCs, and the tumor microenvironment. Our study sheds light on a novel mechanism underlying gemcitabine resistance, focusing on CKS1B and its role in FOXM1-mediated tumorigenesis and chemoresistance.

Our findings demonstrates that CKS1B expression is upregulated during pancreatic carcinogenesis and correlates with poor patient prognosis, aligning with previous reports^23^, ^24^. Analysis of single-cell and RNA-sequencing datasets and our various mouse models revealed progressive CKS1B upregulation from pancreatitis to PDAC, suggesting its potential as a biomarker for early disease detection. Functional studies confirmed that CKS1B promotes cell proliferation, migration, and inhibits apoptosis in PDAC cells, both *in vitro* and *in vivo*. KRAS, CDKN2A, TP53, and SMAD4 are the primary driver oncogenes in PDAC, with KRAS mutations occurring in over 90% of cases^51^. Consequently, extensive efforts have been made to target KRAS therapeutically over the past few decades, although the outcomes have been largely disappointing. Preclinical studies on KRAS^G12D^ inhibitors, such as MRTX1133^52^ and RMC-6236^53^, emphasize the therapeutic potential of KRAS mutations. Notably, upregulation of CKS1B has been observed in early lesions harboring KRAS mutations, suggesting that the association between Kras mutations and CKS1B warrants further investigation. Given that CKS1B expression progressively increases during tumorigenesis and KRAS mutations occur early in this process, KRAS-mutant cells with high CKS1B expression exhibit substantial potential for malignant transformation in PDAC. These findings underscore the potential role of CKS1B in KRAS-driven PDAC development. Further investigation into the CKS1B-KRAS interaction could provide new avenues for early diagnosis and targeted therapies.

Our investigation identified a close association between CKS1B and PDAC CSCs. CSCs was characterized by their capacity for self-renewal and differentiation, play pivotal roles in tumor progression, invasion, and drug resistance^40^, ^54^, ^55^. CKS1B knockdown significantly reduced the self-renewal capacity and number of CSCs, as evidenced by *in vitro* tumor sphere formation, increased CD44^+^CD24^+^/CD133^+^ cell populations, and decreased expression of CSCs markers (ABCG2, C-MYC, ALDH1, BMI-1). These markers are critical as they reflect the reprogramming of cells into CSCs and promoting cellular plasticity, which enables tumor cells to adapt to environmental changes and enhance their survival^40^-^43^. Limiting dilution tumorigenesis experiments further confirmed these findings. Targeting CKS1B may offer a promising strategy to eradicate CSCs and improve treatment outcomes. Gemcitabine is a cornerstone of chemotherapy regimens for PDAC, yet the intricacies of its resistance mechanisms warrant further exploration. Shi *et al.* proposed that CKS1B, as an oncogenic factor, could be considered a drug resistance-inducing gene^22^. Elevated expression of CKS1B has been shown to significantly enhance cellular resistance in multiple cancers, including multiple myeloma^45^, hepatocellular carcinoma^21^, and lung cancer^46^. In pancreatic cancer, Li *et al.*^24^ utilized the pRRophetic algorithm to identify that the IC50 values of common chemotherapeutic agents, such as gemcitabine, 5-FU, and paclitaxel, were lower in patients with high CKS1B expression. This paradox prompted further investigation into the impact of CKS1B expression on chemotherapy sensitivity in pancreatic cancer. Therefore, our study identified a strong association between CKS1B expression and resistance to gemcitabine and oxaliplatin, two DNA synthesis-related drugs commonly used in PDAC treatment according to RNA-sequence dataset. Drug exposure led to increased CKS1B levels, while CKS1B knockdown significantly enhanced PDAC cell sensitivity to these drugs, both *in vivo* and *in vitro*, which is consistent with previous observations that CKS1B-high patients have poor prognosis in other tumors. Published data supports that resistance to apoptosis contributes to increased chemotherapy drug resistance in tumor cells. For instance, Bcl-2 family proteins are known to modulate gemcitabine sensitivity in pancreatic cancer cells^56^, and TIMP1 has been shown to counteract gemcitabine resistance by promoting apoptosis^57^. We observed that CKS1B knockdown combined with gemcitabine treatment significantly enhanced apoptosis and reduced proliferation in PDAC cells, associated with activation of cleaved caspase-3 and caspase-9 both *in vivo* and *in vitro* models. Enhancing apoptosis has been shown to delay gemcitabine resistance^58^, ^59^, suggesting that strategies to promote apoptosis could be effective in treating tumors, either alone or in conjunction with chemotherapy. Furthermore, it is well-documented that CSCs in PDAC modulate tumor cell sensitivity to gemcitabine^60^, ^61^. Our findings also indicate a reduction in stemness protein expression following combined CKS1B knockdown and gemcitabine treatment. In conclusion, consistent with previous studies, CKS1B is recognized as a potential drug resistance gene in various tumors. Our findings further elucidate the mechanism of gemcitabine resistance in pancreatic cancer, demonstrating that CKS1B functions as a key gene in mediating resistance to gemcitabine. It achieves this by promoting apoptosis and enhancing stemness, thereby reducing gemcitabine efficacy. These findings suggest that CKS1B may serve as a prognostic marker in pancreatic cancer and a potential therapeutic target for overcoming drug resistance, offering new opportunities to improve patient survival through combination chemotherapy.

The oncogenic transcription factor FOXM1 is significantly overexpressed in numerous cancer types, including pancreatic, lung adenocarcinoma, and hepatocellular carcinoma. FOXM1 is a well-established regulator of cell cycle progression, proliferation, apoptosis, and other processes critical for tumorigenesis^62^. Dysregulated expression of FOXM1 in pancreatic cancer substantially drives the progression of PDAC^63^, largely due to its role in regulating cell cycle and proliferation^62^. Through its highly conserved sequences in the DNA-binding Forkhead box and C-terminal trans-activation domains, FOXM1 primarily exerts its functions by directly targeting downstream genes^64^. For instance, elevated FOXM1 expression positively regulates glycolysis by transactivating the PDK1 promoter^65^ and promotes hepatocellular carcinoma progression by regulating KIF4A expression^66^. Thus, targeting FOXM1's downstream genes offers a promising avenue for molecular therapies against various malignancies. Wang *et al.* demonstrated that FOXM1 regulates CKS1B expression in osteosarcoma^49^. Given this background, it is intriguing to explore whether FOXM1 regulates CKS1B in pancreatic cancer, potentially contributing to the malignant phenotype of PDAC. Currently, no studies have specifically characterized the role of the FOXM1-CKS1B axis in tumors. Here, we identified a novel regulatory axis wherein FOXM1 directly binds to the CKS1B promoter and upregulates its expression, thereby contributing to the malignant phenotype of PDAC cells. Targeting CKS1B may be clinically significant in counteracting the oncogenic role of FOXM1 and could play an essential role in delaying PDAC progression.

Previous studies have linked high FOXM1 expression in PDAC with poor prognosis and gemcitabine resistance^67^, ^68^. Yan Chen reported that knockdown of FOXM1 with siRNA promotes apoptosis in FaDu cells^69^, while destabilizing the FOXM1 protein induces G2/M cell-cycle arrest and apoptosis and blocks tumor progression^70^, ^71^. Consistent with these findings, our data illustrate that CKS1B contributes to chemoresistance in PDAC by inducing apoptosis. We have identified a novel FOXM1-CKS1B axis capable of regulating multiple malignant biological behaviors, suggesting that the apoptosis-inducing effects of CKS1B may also be mediated under the regulation of FOXM1. Madhi *et al.* reported a positive correlation between FOXM1 and PD-L1 expression in LUAD patients^72^, identifying FOXM1 as a key transcription factor in regulating immune checkpoint inhibitor (ICI) responses^73^. Additionally, Li and colleagues demonstrated that CKS1B knockdown by short hairpin RNA significantly reduced pancreatic cancer cell viability and invasion through regulation of PD-L1 expression^24^. Consequently, further investigation is warranted to explore whether the FOXM1-CKS1B axis regulates PD-L1 expression, which could open new avenues in immunotherapy. In conclusion, our newly identified FOXM1-CKS1B axis represents a promising therapeutic target for PDAC intervention.

In recent years, research on the antitumor effects of FOXM1 inhibitors has been actively pursued. Derivatives of natural compounds, including casticin and honokiol, have shown the ability to suppress FOXM1 activity and its downstream effector molecules. Through high-throughput screening, compounds such as RCM1, FDI-6, 9R-201, NB-55, NB-73, and NB-115 have been identified as highly specific FOXM1 inhibitors^74^. However, no FOXM1 inhibitors have yet entered clinical trials, underscoring the urgent need for in-depth mechanistic studies of FOXM1 to develop safer and more effective therapeutic agents. Our research demonstrates that the FOXM1-CKS1B axis plays a critical role in various malignant biological phenotypes of pancreatic cancer, including tumorigenesis, proliferation, migration, stemness, and chemoresistance. Thus, targeting the FOXM1-CKS1B axis presents a promising therapeutic approach, offering new directions for the development of FOXM1 inhibitors.

Our study has limitations. Future clinical trials are necessary to validate our observations and translate them into clinical applications. Additionally, the specific roles of CKS1B in immune infiltration and autophagy in PDAC require further investigation^24^. Unraveling these mechanisms will provide a more comprehensive understanding of CKS1B's role in PDAC and pave the way for the development of more effective treatment strategies.

In conclusion, our study unveiled the functional significance of CKS1B throughout PDAC development. CKS1B played a crucial role in promoting cell proliferation, migration, and stemness in PDAC, ultimately contributing to tumor progression and chemoresistance. The CKS1B-FOXM1 axis represents a promising therapeutic target for PDAC, offering potential avenues for improving treatment outcomes. Further research is warranted to fully elucidate the mechanisms underlying CKS1B's role in PDAC and to develop effective strategies targeting this pathway.

## Materials and Methods

### Cell lines and culture

Human pancreatic ductal acinar cell lines BxPC-3, CFPAC-1, MIA PaCa-2, PANC-1, Panc02 and PL45 were obtained from Procell Life Science & Technology. BxPC-3 and Panc02 cells were cultured in RPMI 1640 (Gibco), while CFPAC-1 cells were cultured in IMDM (Gibco). PANC1, MIA PaCa-2 and PL45 cells were cultured in DMEM (Gibco). All media were supplemented with 10% fetal bovine serum (FBS) and 1% penicillin/streptomycin. Cells were maintained at 37°C in a humidified chamber with 5% CO2. Cells were passaged with 0.25% trypsin/2.21 mM EDTA in PBS when they reached 90% confluency. All the cell lines were obtained between 2020 and 2022, routinely tested for mycoplasma contamination within the last 6 months by using PCR, and used at passage numbers <15 for this study after reception or thawing in our laboratory.

### Cell proliferation assay

Cell proliferation was assessed using the Cell Counting Kit-8 (CCK-8) assay. Briefly, 1000 cells were seeded in 100 μL of medium per well of a 96-well plate. After transfection with siRNA according to the manufacturer's protocol, the medium was changed to a low-serum medium. After 24, 48, 72 and 96 h, 10 μL of CCK-8 solution was added to each well, and the plate was incubated for 1 hour at 37°C. Absorbance at 450 nm was measured using a microplate reader.

For the drug-resistance assay, BxPC-3 and CFPAC-1 cells were treated with different concentrations of gemcitabine. The absorbance at 450 nm was measured after 48 hours of incubation.

### Colony formation assay

Colony formation was measured to assess cell proliferation potential. A total of 2500 cells were seeded per well in a six-well plate and cultured for 2 weeks. The medium was changed every 4 days. Colonies were fixed with methanol for 20 minutes and stained with 0.1% crystal violet. The number of colonies was counted after staining.

### Cell migration assay

A wound-healing assay was used to assess cell migration ability. Cells were seeded in a 6-well plate and transfected with siRNA according to the manufacturer's protocol. Once the cells reached 90% confluency, a sterile 200 μL pipette tip was used to scratch three wounds through the cell monolayer. Cells were washed with PBS to remove detached cells and incubated for 0, 12, and 24 h. Images were captured using an inverted microscope.

Transwell assay was assessed to investigate cell migrant. 2×10^4^ cells were seeded onto the top chambers of 8 μm pore-size transwell inserts. After 48 h, invaded cells were fixed with methanol for 20 min and stained with 0.1% crystal violet. The number of invaded cells was counted after staining.

### Tumor sphere formation assay

Tumor sphere formation was assessed to evaluate cancer stem cell activity. 1×10^4^ cells were seeded per well in ultralow attachment six-well plates and cultured in serum-free DMEM/F12 supplemented with EGF (20 ng/mL), bFGF (20 ng/mL), and B27 (1:50). After 5-7 days, formed tumor spheres were counted under a phase-contrast microscope.

### Cell cycle and apoptosis analysis

Cells were seeded in 6-well plates at a density of 5×10^5^ cells per well. For cell cycle analysis, cells were harvested after 48 h of transfection, fixed in 75% ethanol at 4°C overnight, and stained with PI/RNase A solution using the Cell Cycle and Apoptosis Analysis Kit according to the manufacturer's instructions. For apoptosis analysis, cells were stained with FITC Annexin V and PI using the Annexin V-FITC Cell Apoptosis Detection Kit according to the manufacturer's instructions. Cell cycle distribution and apoptosis were analyzed by flow cytometry.

### Chemotherapy Resistance Analysis

To investigate the relationship between CKS1B expression and chemotherapy resistance, we trained elastic net regression models using the oncoPredict R package^75^, based on public drug response data from GDSC (Genomics of Drug Sensitivity in Cancer) and CCLE (Cancer Cell Line Encyclopedia). These models were applied to TCGA-PAAD expression data (from UCSC XENA) to identify correlations between CKS1B expression and drug sensitivity, with IC50 as the measure. The calPhenotype function was used to calculate the correlation between CKS1B expression and sensitivity to oxaliplatin and gemcitabine. Patients were then divided into high and low CKS1B expression groups, and t-tests were used to compare drug sensitivity between the two groups.

### RNA isolation and RT-PCR

Total RNA was extracted from cells using a RNAiso Plus reagent. Then cDNA was synthesized using the PrimeScript RT reagent kit according to the manufacturer's instructions. Primer sequences are listed in the Supplementary Materials. The fold change in gene expression was calculated using the 2^-ΔΔCt^ method.

### Animal experiments

All animal experiments were conducted in accordance with the animal experimental guidelines of the South China University of Technology and approved by their Institutional Animal Care and Use Committee. Mice were housed under specific pathogen-free conditions with a 12-h light/dark cycle, temperature of 22°C, and 55% humidity.

### Xenograft mouse models

Six to eight-week-old male NOD/SCID mice received subcutaneous injections of 1×10^6^ CFPAC-1 cells suspended in 100 μL PBS mixed with Matrigel (1:1) per flank. Once tumors reached 50-100 mm³, mice were randomly divided into four groups (n= 5-6): (1) negative control siRNA, (2) CKS1B-siRNA, (3) negative control + gemcitabine, (4) CKS1B-siRNA + gemcitabine. Groups 2 and 4 received intratumoral injections of *in vivo*-jet PEI Delivery Kit and CKS1B siRNA (negative control) every 3 days to knock down CKS1B expression. Groups 1 and 3 received *in vivo*-jet PEI with negative control siRNA. Gemcitabine (50 mg/kg) or saline was administered intraperitoneally every 3 days.

To evaluate tumor growth, six to eight-week-old male C57BL/6 mice were utilized. CKS1B-overexpressing Panc02 cells or vector control (pc3.1) cells were prepared at various concentrations in a 1:1 mixture of PBS and Matrigel, these suspensions were subcutaneously injected into the left (control group) and right (experimental group) dorsal thighs of each mouse.

Tumor volume was measured and calculated using a Vernier caliper and formula (0.5×length×width^2^) every 3 days. Mice were sacrificed at the end of the experiment, and subcutaneous tumors were collected for further analysis.

### Intratumoral injection of siRNAs

The siRNA formulation for intratumoral delivery using *in vivo*-jet PEI was prepared according to the manufacturer's instructions. Briefly, 5 μg of siRNAs diluted in a sterile 5% glucose solution were complexed with *in vivo*-jet PEI at a ratio of 0.12 μL per 1 μg siRNA. Formulated siRNAs were injected intratumorally twice a week.

### Pancreatitis and precancerous mouse lesions models

The primary cell line 266-6 was seeded one day prior to experimentation. RNA was collected after 24 h of induction with 50 nM TGF-α.

Six-to eight-week-old C57BL/6 mice (mixed male and female) were used for pancreatitis and precancerous lesion models. Pancreatitis was induced by intraperitoneal injections of 2 g/kg L-arginine. Retrograde ductal infusion-induced acute pancreatitis was induced by infusing 2 mg/g 2% sodium taurocholate into the pancreatic duct. Mouse pancreata were harvested at specified time points.

Precancerous lesions were induced by intraperitoneal injections of 80 μg/kg caerulein in 6-8 week old mice. Pancreatic duct ligation (PDL) models were created by anesthetizing mice and surgically exposing the pancreas. The pancreatic duct was ligated with a 7-0 non-absorbable suture. Pancreata were collected for immunolabeling at designated time points.

### TUNEL assay

CFPAC-1 tumors treated with gemcitabine combination therapy were processed for paraffin sections and TUNEL staining using the TUNEL Apoptosis Detection Kit according to the manufacturer's instructions.

### CHIP assay

Chromatin immunoprecipitation (ChIP) assays were performed using the Simple ChIP Plus Enzymatic Chromatin IP Kit according to the manufacturer's instructions. Chromatin was immunoprecipitated with anti-FOXM1 antibodies. ChIP-derived DNA was quantified using quantitative RT-PCR with primers listed in the Supplemental Materials.

### Luciferase reporter assay

The CKS1B promoter sequence (-400 to 100, 500 bp) was synthesized and inserted into the multiple cloning site (*Kpn I* and *Nhe I*) of the PGL3-Basic vector (Promega, USA). The FoxM1 binding site 1 (BS1) was mutated to “GGACCCCCCCGA” and FoxM1 binding site 2 (BS2) was mutated to “TGGCCCCCCCGA”. HEK293 cells were seeded into 24-well cell culture plates at 5×10^4^ cells per well. The cells were co-transfected with 100 ng of reporter gene vector and 100 ng of FoxM1-pcDNA3.1 using Lipofectamine 2000. pcDNA3.1 was used as a control. Cells were collected at 30 h after transfection to perform Renilla luciferase activity assay (Promega, USA). The fluorescence activity was normalized by protein content.

### Immunohistochemical analysis (IHC)

Formalin-fixed, paraffin-embedded sections (3.5 µm) were deparaffinized and rehydrated. Subsequent steps included heat-induced epitope retrieval with citrate buffer (pH 6.0) or Tris/EDTA (pH 9.0), endogenous peroxidase blocking with 3% H_2_O_2_, and non-specific protein blocking with 10% goat serum. Overnight incubation with specific primary antibodies (detailed in **Supplementary Tables**) followed. After thorough washes in TBST, sections were incubated with biotinylated secondary antibody, exposed to diaminobenzidine, and counterstained with Hematoxylin. Finally, after serial dehydration, slides were mounted for microscopic examination.

### Multiplex immunohistochemistry staining

Multiplex immunohistochemistry (mIHC) was performed using the PANO Multiplex IHC kit (Panovue, 10144100100). Formalin-fixed paraffin-embedded sections (3.5 µm) were deparaffinized and rehydrated. Each slide underwent several cycles of staining, including heat-induced epitope retrieval with Tris/EDTA (pH=9.0), endogenous peroxidase blocking with 3% H_2_O_2_, and non-specific protein blocking with 10% goat serum, followed by incubation of primary antibodies and corresponding horseradish peroxidase-conjugated secondary antibody (Panovue, 10013001040). Finally, tyramide signal amplification dyes were applied to amplify fluorescence signals. The following primary antibodies were used in sequential rounds of staining: CKS1B (Invitrogen, Cat#36-6800, 1:100), CK19 (Abcam, Cat#ab52625, 1:500), amylase (Santa, Cat#sc-46657, 1:500). Nuclei were counterstained with DAPI, and coverslips were mounted with antifade mountant.

### Immunofluorescence

Cultured BxPC-3 and CFPAC-1 cells were fixed with 4% paraformaldehyde formalin for 20 min at room temperature. Tumor tissue sections (3.5 µm thick) underwent deparaffinization, rehydration, and antigen retrieval (citrate buffer, pH 6.0 or Tris/EDTA, pH 9.0). Both fixed cells and antigen-retrieved tissues were permeabilized with 0.1% Triton X-100, followed by incubation with blocking buffer (5% BSA in TBS containing 0.025% Triton X-100) for 1 h. Overnight incubation with the appropriate primary antibodies (detailed in **Supplementary Tables**) was performed. Corresponding fluorochrome-conjugated (Alexa Fluor 488) secondary antibodies diluted in TBST containing 5% BSA (1:1000) were subsequently used. Nuclei were counterstained with DAPI (50 ng/mL), and coverslips were mounted with antifade mountant. Visualization of fluorescence was achieved under a fluorescence microscope.

### Western blot analysis

Cell lysates were prepared using SDS lysis buffer, and protein samples were separated by 12% SDS-PAGE gels and transferred to 0.22 µm PVDF membranes. Membranes were blocked with 5% milk at room temperature. Subsequent incubation with primary antibodies, followed by horseradish peroxidase (HRP)-conjugated secondary antibodies, was performed. Immunoreactive protein bands were detected using a chemiluminescence solution. Protein lysates were subjected to electrophoresis using a methodology reported in the literature^76^, and images were automatically captured using the ProteinSimple system equipped with a 25-capillary cartridge (ranging from 2 to 40 kDa), EZ Standard Biotinylated Ladder, and Detection Module (ProteinSimple Japan Co. Ltd., Chuo-ku, Tokyo, Japan). Analysis of target protein bands was performed using Compass software provided by ProteinSimple Japan Co. Ltd. The utilized antibodies are listed in **Supplementary Tables**.

## Supplementary Material

Supplementary figures and tables.

## References

[1] Zeng S, Pöttler M, Lan B, Grützmann R, Pilarsky C, Yang H (2019). Chemoresistance in Pancreatic Cancer. International journal of molecular sciences.

[2] Wood LD, Canto MI, Jaffee EM, Simeone DM (2022). Pancreatic Cancer: Pathogenesis, Screening, Diagnosis, and Treatment. Gastroenterology.

[3] Matthews HK, Bertoli C, de Bruin RAM (2022). Cell cycle control in cancer. Nat Rev Mol Cell Biol.

[4] Williams GH, Stoeber K (2012). The cell cycle and cancer. J Pathol.

[5] Suski JM, Braun M, Strmiska V, Sicinski P (2021). Targeting cell-cycle machinery in cancer. Cancer Cell.

[6] Pribnow A, Jonchere B, Liu J, Smith KS, Campagne O, Xu K (2022). Combination of Ribociclib and Gemcitabine for the Treatment of Medulloblastoma. Molecular cancer therapeutics.

[7] Montopoli M, Ragazzi E, Froldi G, Caparrotta L (2009). Cell-cycle inhibition and apoptosis induced by curcumin and cisplatin or oxaliplatin in human ovarian carcinoma cells. Cell Prolif.

[8] Krishnan A, Nair SA, Pillai MR (2010). Loss of cks1 homeostasis deregulates cell division cycle. Journal of cellular and molecular medicine.

[9] Mu R, Tat J, Zamudio R, Zhang Y, Yates JR, Kumagai A (2017). CKS Proteins Promote Checkpoint Recovery by Stimulating Phosphorylation of Treslin. Molecular and cellular biology.

[10] Westbrook L, Manuvakhova M, Kern FG, Estes NR, Ramanathan HN, Thottassery JV (2007). Cks1 regulates cdk1 expression: a novel role during mitotic entry in breast cancer cells. Cancer research.

[11] Tang Y, Reed SI (1993). The Cdk-associated protein Cks1 functions both in G1 and G2 in Saccharomyces cerevisiae. Genes & development.

[12] Shapira Ma, Ben-Izhak O, Bishara B, Futerman B, Minkov I, Krausz MM (2004). Alterations in the expression of the cell cycle regulatory protein cyclin kinase subunit 1 in colorectal carcinoma. Cancer.

[13] Koga H, Harada M, Ohtsubo M, Shishido S, Kumemura H, Hanada S (2003). Troglitazone induces p27Kip1-associated cell-cycle arrest through down-regulating Skp2 in human hepatoma cells. Hepatology.

[14] Tsai Y-S, Chang H-C, Chuang L-Y, Hung W-C (2005). RNA silencing of Cks1 induced G2/M arrest and apoptosis in human lung cancer cells. IUBMB life.

[15] Scaife RM (2004). G2 cell cycle arrest, down-regulation of cyclin B, and induction of mitotic catastrophe by the flavoprotein inhibitor diphenyleneiodonium. Molecular cancer therapeutics.

[16] Zhan F, Colla S, Wu X, Chen B, Stewart JP, Kuehl WM (2007). CKS1B, overexpressed in aggressive disease, regulates multiple myeloma growth and survival through SKP2- and p27Kip1-dependent and -independent mechanisms. Blood.

[17] Lin L, Fang Z, Lin H, You H, Wang J, Su Y (2016). Depletion of Cks1 and Cks2 expression compromises cell proliferation and enhance chemotherapy-induced apoptosis in HepG2 cells. Oncology reports.

[18] Zeng Z, Gao Z-L, Zhang Z-P, Jiang H-B, Yang C-Q, Yang J (2019). Downregulation of CKS1B restrains the proliferation, migration, invasion and angiogenesis of retinoblastoma cells through the MEK/ERK signaling pathway. International journal of molecular medicine.

[19] Slotky M, Shapira Ma, Ben-Izhak O, Linn S, Futerman B, Tsalic M (2005). The expression of the ubiquitin ligase subunit Cks1 in human breast cancer. Breast cancer research: BCR.

[20] Wang X, Xu J, Ju S, Ni H, Zhu J, Wang H (2010). Livin gene plays a role in drug resistance of colon cancer cells. Clin Biochem.

[21] Lee E-K, Kim D-G, Kim J-S, Yoon Y (2011). Cell-cycle regulator Cks1 promotes hepatocellular carcinoma by supporting NF-κB-dependent expression of interleukin-8. Cancer research.

[22] Shi W, Huang Q, Xie J, Wang H, Yu X, Zhou Y (2020). CKS1B as Drug Resistance-Inducing Gene-A Potential Target to Improve Cancer Therapy. Frontiers in oncology.

[23] Tang Y, Lan X, Yan M, Fu Z, Li H (2024). CKS1B as a potential target for prognostic assessment and intervention in pancreatic cancer and its role in abnormal proliferation and cellular phenotype through mediation of cell cycle signaling pathways. Saudi Med J.

[24] Li L, Wang J, Zhang Z, Yang Q, Deng Z, Zou W (2022). Identification of CKS1B as a prognostic indicator and a predictive marker for immunotherapy in pancreatic cancer. Frontiers in immunology.

[25] Safa AR (2022). Drug and apoptosis resistance in cancer stem cells: a puzzle with many pieces. Cancer Drug Resist.

[26] Sancho P, Alcala S, Usachov V, Hermann PC, Sainz B (2016). The ever-changing landscape of pancreatic cancer stem cells. Pancreatology.

[27] Kalin TV, Ustiyan V, Kalinichenko VV (2011). Multiple faces of FoxM1 transcription factor: lessons from transgenic mouse models. Cell cycle (Georgetown, Tex).

[28] Liao G-B, Li X-Z, Zeng S, Liu C, Yang S-M, Yang L (2018). Regulation of the master regulator FOXM1 in cancer. Cell Commun Signal.

[29] Arceci A, Bonacci T, Wang X, Stewart K, Damrauer JS, Hoadley KA (2019). FOXM1 Deubiquitination by USP21 Regulates Cell Cycle Progression and Paclitaxel Sensitivity in Basal-like Breast Cancer. Cell Rep.

[30] Quan M, Wang P, Cui J, Gao Y, Xie K (2013). The roles of FOXM1 in pancreatic stem cells and carcinogenesis. Mol Cancer.

[31] Li Z, Jia Z, Gao Y, Xie D, Wei D, Cui J (2015). Activation of vitamin D receptor signaling downregulates the expression of nuclear FOXM1 protein and suppresses pancreatic cancer cell stemness. Clinical cancer research: an official journal of the American Association for Cancer Research.

[32] Li L, Li Z, Kong X, Xie D, Jia Z, Jiang W (2014). Down-regulation of microRNA-494 via loss of SMAD4 increases FOXM1 and β-catenin signaling in pancreatic ductal adenocarcinoma cells. Gastroenterology.

[33] Li Q, Zhang N, Jia Z, Le X, Dai B, Wei D (2009). Critical role and regulation of transcription factor FoxM1 in human gastric cancer angiogenesis and progression. Cancer research.

[34] Sendler M, van den Brandt C, Glaubitz J, Wilden A, Golchert J, Weiss FU (2020). NLRP3 Inflammasome Regulates Development of Systemic Inflammatory Response and Compensatory Anti-Inflammatory Response Syndromes in Mice With Acute Pancreatitis. Gastroenterology.

[35] Qi-Xiang M, Yang F, Ze-Hua H, Nuo-Ming Y, Rui-Long W, Bin-Qiang X (2022). Intestinal TLR4 deletion exacerbates acute pancreatitis through gut microbiota dysbiosis and Paneth cells deficiency. Gut Microbes.

[36] Laukkarinen JM, Van Acker GJD, Weiss ER, Steer ML, Perides G (2007). A mouse model of acute biliary pancreatitis induced by retrograde pancreatic duct infusion of Na-taurocholate. Gut.

[37] Perides G, van Acker GJD, Laukkarinen JM, Steer ML (2010). Experimental acute biliary pancreatitis induced by retrograde infusion of bile acids into the mouse pancreatic duct. Nat Protoc.

[38] Feng Y, Cai L, Pook M, Liu F, Chang C-H, Mouti MA (2024). BRD9-SMAD2/3 Orchestrates Stemness and Tumorigenesis in Pancreatic Ductal Adenocarcinoma. Gastroenterology.

[39] Hermann PC, Sainz B (2018). Pancreatic cancer stem cells: A state or an entity?. Semin Cancer Biol.

[40] Nagaraju GP, Farran B, Luong T, El-Rayes BF (2023). Understanding the molecular mechanisms that regulate pancreatic cancer stem cell formation, stemness and chemoresistance: A brief overview. Semin Cancer Biol.

[41] Zhao Y, Qin C, Zhao B, Wang Y, Li Z, Li T (2023). Pancreatic cancer stemness: dynamic status in malignant progression. Journal of experimental & clinical cancer research: CR.

[42] Bubin R, Uljanovs R, Strumfa I (2023). Cancer Stem Cells in Pancreatic Ductal Adenocarcinoma. International journal of molecular sciences.

[43] Zhou J, Wang H, Che J, Xu L, Yang W, Li Y (2020). Silencing of microRNA-135b inhibits invasion, migration, and stemness of CD24+CD44+ pancreatic cancer stem cells through JADE-1-dependent AKT/mTOR pathway. Cancer Cell Int.

[44] Liu Y, Clem B, Zuba-Surma EK, El-Naggar S, Telang S, Jenson AB (2009). Mouse fibroblasts lacking RB1 function form spheres and undergo reprogramming to a cancer stem cell phenotype. Cell Stem Cell.

[45] Shi L, Wang S, Zangari M, Xu H, Cao TM, Xu C (2010). Over-expression of CKS1B activates both MEK/ERK and JAK/STAT3 signaling pathways and promotes myeloma cell drug-resistance. Oncotarget.

[46] Wang H, Sun M, Guo J, Ma L, Jiang H, Gu L (2017). 3-O-(Z)-coumaroyloleanolic acid overcomes Cks1b-induced chemoresistance in lung cancer by inhibiting Hsp90 and MEK pathways. Biochemical pharmacology.

[47] Mohammad RM, Muqbil I, Lowe L, Yedjou C, Hsu H-Y, Lin L-T (2015). Broad targeting of resistance to apoptosis in cancer. Semin Cancer Biol.

[48] Carneiro BA, El-Deiry WS (2020). Targeting apoptosis in cancer therapy. Nat Rev Clin Oncol.

[49] Wang IC, Chen Y-J, Hughes D, Petrovic V, Major ML, Park HJ (2005). Forkhead box M1 regulates the transcriptional network of genes essential for mitotic progression and genes encoding the SCF (Skp2-Cks1) ubiquitin ligase. Molecular and cellular biology.

[50] Zhang J, Wang Y, Wang L, You L, Zhang T (2024). Pancreatic ductal adenocarcinoma chemoresistance: From metabolism reprogramming to novel treatment. Chin Med J (Engl).

[51] Hu ZI, O'Reilly EM (2024). Therapeutic developments in pancreatic cancer. Nat Rev Gastroenterol Hepatol.

[52] Wei D, Wang L, Zuo X, Maitra A, Bresalier RS (2024). A Small Molecule with Big Impact: MRTX1133 Targets the KRASG12D Mutation in Pancreatic Cancer. Clinical cancer research: an official journal of the American Association for Cancer Research.

[53] Gustafson WC, Wildes D, Rice MA, Lee BJ, Jiang J, Wang Z (2022). Direct targeting of RAS in pancreatic ductal adenocarcinoma with RMC-6236, a first-in-class, RAS-selective, orally bioavailable, tri-complex RASMULTI (ON) inhibitor. American Society of Clinical Oncology.

[54] Kahroba H, Shirmohamadi M, Hejazi MS, Samadi N (2019). The Role of Nrf2 signaling in cancer stem cells: From stemness and self-renewal to tumorigenesis and chemoresistance. Life Sci.

[55] Nunes T, Hamdan D, Leboeuf C, El Bouchtaoui M, Gapihan G, Nguyen TT (2018). Targeting Cancer Stem Cells to Overcome Chemoresistance. International journal of molecular sciences.

[56] Bauer C, Hees C, Sterzik A, Bauernfeind F, Mak'Anyengo R, Duewell P (2015). Proapoptotic and antiapoptotic proteins of the Bcl-2 family regulate sensitivity of pancreatic cancer cells toward gemcitabine and T-cell-mediated cytotoxicity. J Immunother.

[57] Tan Y, Li X, Tian Z, Chen S, Zou J, Lian G (2021). TIMP1 down-regulation enhances gemcitabine sensitivity and reverses chemoresistance in pancreatic cancer. Biochemical pharmacology.

[58] Zechner D, Albert A-C, Bürtin F, Vollmar B (2017). Metformin Inhibits Gemcitabine Induced Apoptosis in Pancreatic Cancer Cell Lines. J Cancer.

[59] Jin J, Xiong Y, Cen B (2017). Bcl-2 and Bcl-xL mediate resistance to receptor tyrosine kinase-targeted therapy in lung and gastric cancer. Anticancer Drugs.

[60] Nachiyappan A, Gupta N, Taneja R (2022). EHMT1/EHMT2 in EMT, cancer stemness and drug resistance: emerging evidence and mechanisms. FEBS J.

[61] Xia S, Pan Y, Liang Y, Xu J, Cai X (2020). The microenvironmental and metabolic aspects of sorafenib resistance in hepatocellular carcinoma. EBioMedicine.

[62] Huang C, Du J, Xie K (2014). FOXM1 and its oncogenic signaling in pancreatic cancer pathogenesis. Biochimica et biophysica acta.

[63] Liu W, Tang J, Zhang H, Kong F, Zhu H, Li P (2020). A novel lncRNA PTTG3P/miR-132/212-3p/FoxM1 feedback loop facilitates tumorigenesis and metastasis of pancreatic cancer. Cell death discovery.

[64] Nandi D, Cheema PS, Jaiswal N, Nag A (2018). FoxM1: Repurposing an oncogene as a biomarker. Semin Cancer Biol.

[65] Yang Q, Wu F, Zhang Y, Wang R (2022). FOXM1 regulates glycolysis in nasopharyngeal carcinoma cells through PDK1. Journal of cellular and molecular medicine.

[66] Hu G, Yan Z, Zhang C, Cheng M, Yan Y, Wang Y (2019). FOXM1 promotes hepatocellular carcinoma progression by regulating KIF4A expression. Journal of experimental & clinical cancer research: CR.

[67] Liu C, Shi J, Li Q, Li Z, Lou C, Zhao Q (2019). STAT1-mediated inhibition of FOXM1 enhances gemcitabine sensitivity in pancreatic cancer. Clin Sci (Lond).

[68] Roca MS, Moccia T, Iannelli F, Testa C, Vitagliano C, Minopoli M (2022). HDAC class I inhibitor domatinostat sensitizes pancreatic cancer to chemotherapy by targeting cancer stem cell compartment via FOXM1 modulation. Journal of experimental & clinical cancer research: CR.

[69] Chen Y, Liu Y, Ni H, Ding C, Zhang X, Zhang Z (2017). FoxM1 overexpression promotes cell proliferation and migration and inhibits apoptosis in hypopharyngeal squamous cell carcinoma resulting in poor clinical prognosis. International journal of oncology.

[70] Nandi I, Smith HW, Sanguin-Gendreau V, Ji L, Pacis A, Papavasiliou V (2023). Coordinated activation of c-Src and FOXM1 drives tumor cell proliferation and breast cancer progression. J Clin Invest.

[71] Nestal de Moraes G, Delbue D, Silva KL, Robaina MC, Khongkow P, Gomes AR (2015). FOXM1 targets XIAP and Survivin to modulate breast cancer survival and chemoresistance. Cell Signal.

[72] Madhi H, Lee J-S, Choi YE, Li Y, Kim MH, Choi Y (2022). FOXM1 Inhibition Enhances the Therapeutic Outcome of Lung Cancer Immunotherapy by Modulating PD-L1 Expression and Cell Proliferation. Adv Sci (Weinh).

[73] Tang B, Yan R, Zhu J, Cheng S, Kong C, Chen W (2022). Integrative analysis of the molecular mechanisms, immunological features and immunotherapy response of ferroptosis regulators across 33 cancer types. Int J Biol Sci.

[74] Khan MA, Khan P, Ahmad A, Fatima M, Nasser MW (2023). FOXM1: A small fox that makes more tracks for cancer progression and metastasis. Semin Cancer Biol.

[75] Maeser D, Gruener RF, Huang RS (2021). oncoPredict: an R package for predicting *in vivo* or cancer patient drug response and biomarkers from cell line screening data. Brief Bioinform.

[76] Nguyen U, Squaglia N, Boge A, Fung PA (2011). The Simple Western™: a gel-free, blot-free, hands-free Western blotting reinvention. Nature Methods.

