# Supplementary material for: FOXM1-Driven CKS1B Upregulation Promotes Pancreatic Cancer Progression and Therapeutic Resistance
Source: Int J Biol Sci. 2025 Jan 13;21(3):1047–64. doi: 10.7150/ijbs.105289 (PMC11781179; doi:10.7150/ijbs.105289)
Supplement: Supplementary file 1 — Supplementary figures and tables. [file ijbsv21p1047s1.pdf]

Supplementary Information

Antibodies used in the study		
**Antigen**	**Company**	**No.**
**CKS1B**	Invitrogen	36-6800
**Cleaved-caspase3**	Cell Signal Technology	20750S
**Cleaved-caspase9**	Cell Signal Technology	9661
**Ki67**	Abcam	ab16667
**PCNA**	Proteintech	60097-1-Ig
**CD44**	Cell Signal Technology	#3570
**FOXM1**	Cell Signal Technology	20459
**FOXM1**	Cell Signal Technology	5436S
**OCT-4**	BIOWORLD	BS70993
**SOX2**	Proteintech	66411-1-Ig
**BMI-1**	BIOWORLD	MB9014
**ABCG2**	proteintech	27286-1-AP
**ALDH1**	ABCAM	ab23375
**C-MYC**	Cell Signal Technology	9402
**Cyclin D1**	Proteintech	26939-1-AP
**P21**	Proteintech	10355-1-AP
**P27;KIP1**	Proteintech	25614-1-AP
**CD44**	BioLegend	338803
**CD24**	BioLegend	311105
**CD133**	BioLegend	372803
**CK19**	abcam	ab52625
**Amylase**	Santa	sc-46657

PCR primers used in the study		
**HUMAN**		
**Gene**	**Forward**	**Reverse**
**CKS1B**	TATTCGGACAAATACGACGACG	CGCCAAGATTCCTCCATTCAGA
**FOXM1**	TGCAGCTAGGGATGTGAATCTTC	GGAGCCCAGTCCATCAGAACT
**GAPDH**	GCGAGATCCCTCCAAAATCA	ACTTCTCATGGTTCACACCC
**BMI-1**	CGTGTATTGTTCGTTACCTGGA	TTCAGTAGTGGTCTGGTCTTGT
**ABCG2**	CAGGTGGAGGCAAATCTTCGT	ACCCTGTTAATCCGTTCGTTTT
**ALDH1**	CCGTGGCGTACTATGGATGC	GCAGCAGACGATCTCTTTCGAT
**Oct4**	CTTGCTGCAGAAGTGGGTGGAGGAA	CTGCAGTGTGGGTTTCGGGCA
**CD24**	CTCCTACCCACGCAGATTTATTC	AGAGTGAGACCACGAAGAGAC
**MYC**	GGCTCCTGGCAAAAGGTCA	CTGCGTAGTTGTGCTGATGT
**Amylase**	AATACACAACAAGGACGGACATC	TCCAAATCCCTTCGGAGCTAAA
**CK19**	AACGGCGAGCTAGAGGTGA	GGATGGTCGTGTAGTAGTGGC
**MOUSE**		
**Gene**	**Forward**	**Reverse**
**18SRNA**	ATAAACGATGCCGACTGGCGA	AAATTAAGCCGCAGGCCCAC
**CK19**	ATTGGGTCAGGGGGTGTTTT	TGTCCAAGTAGGAGGCGAGA
**AMY**	TGCCAAGGAATGTGAGCGAT	TCCACAGGTACTGCTTGTTCC
**CKS1B**	CTGGTCCCGAAAACCCATCT	AAGAAAGCAACATGGTCACGC
**FOXM1**	GAAAGATGAGTTCTGACGGGCT	CTCAGTGCTGTTGATGGCAA
**GAPDH**	AAGGTGGTGAAGCAGGCATCTGAG	GGAAGAGTGGGAGTTGCTGTTGAAGTC
**Amylase**	TGCCAAGGAATGTGAGCGAT	TCCACAGGTACTGCTTGTTCC

CHIP-primer used in the study		
	**Forward**	**Reverse**
**BS1**	AGGCTTGAAAGTAGAGGTGTCC	TGGCCTCGCTTTCAAACACG
**BS2**	GCTTCCCACTTTCCCGAGTT	AAGAACGAGCGTGGGTCAAA

Reagents used in the study		
**Name**	**Company**	**No.**
TUNEL Apoptosis Detection Kit	Elabscience	E-CK-A320
Simple ChIP Plus Enzymatic Chromatin IP Kit	Invitrogen	91820
the Cell Cycle and Apoptosis Analysis Kit	Beyotime	C1052
the Annexin V-FITC Cell Apoptosis Detection Kit	Beyotime	C1062L
the PrimeScript RT reagent kit	Takara	RR047A
FBS (fetal bovine serum)	Nobimpex	A115-500
RPMI-1640	Gibco	C11875500BT
DMEM	Gibco	C11995500BT
IMDM	Gibco	C12440500BT
Cell Counting Kit-8 (CCK-8)	Dojindo	CK04
Transwell	CORNING	3422
EGF	Peprotech	AF-100-15
bFGF	Peprotech	100-18B
B27	Gibco	2389185
DMEM/F12	Gibco	11320033
gemcitabine	MCE	HY-17026
oxaliplatin	MCE	HY-17371
RNAiso Plus	Takara	9108
In vivo-jet PEI	Polyplus	101000040
L-arginine	Solarbio	A0013
Caerulein	MCE	HY-A0190
2% sodium taurocholate	Merck	S0900000
DAPI	abcam	ab104139
TGF-α	R&D	239-A-500

siRNA used in the study		
	**sense（5'-3'）**	**antisense（5'-3'）**
**CKS1B-1**	GGAGUUUGAGUAUCGACAUTT	AUGUCGAUACUCAAACUCCTT
**CKS1B-2**	CCAUCUGAUGUCUGAAUCUTT	AGAUUCAGACAUCAGAUGGTT
**H-FOXM1**	GGACCACUUUCCCUACUUUtt	AAAGUAGGGAAAGUGGUCCtt
**M-FOXM1**	CCAUUCACCCAAGUGCCAAtt	UUGGCACUUGGGUGAAUGGtt

Clinical characteristics of pancreatic cancer patients from the TCGA database

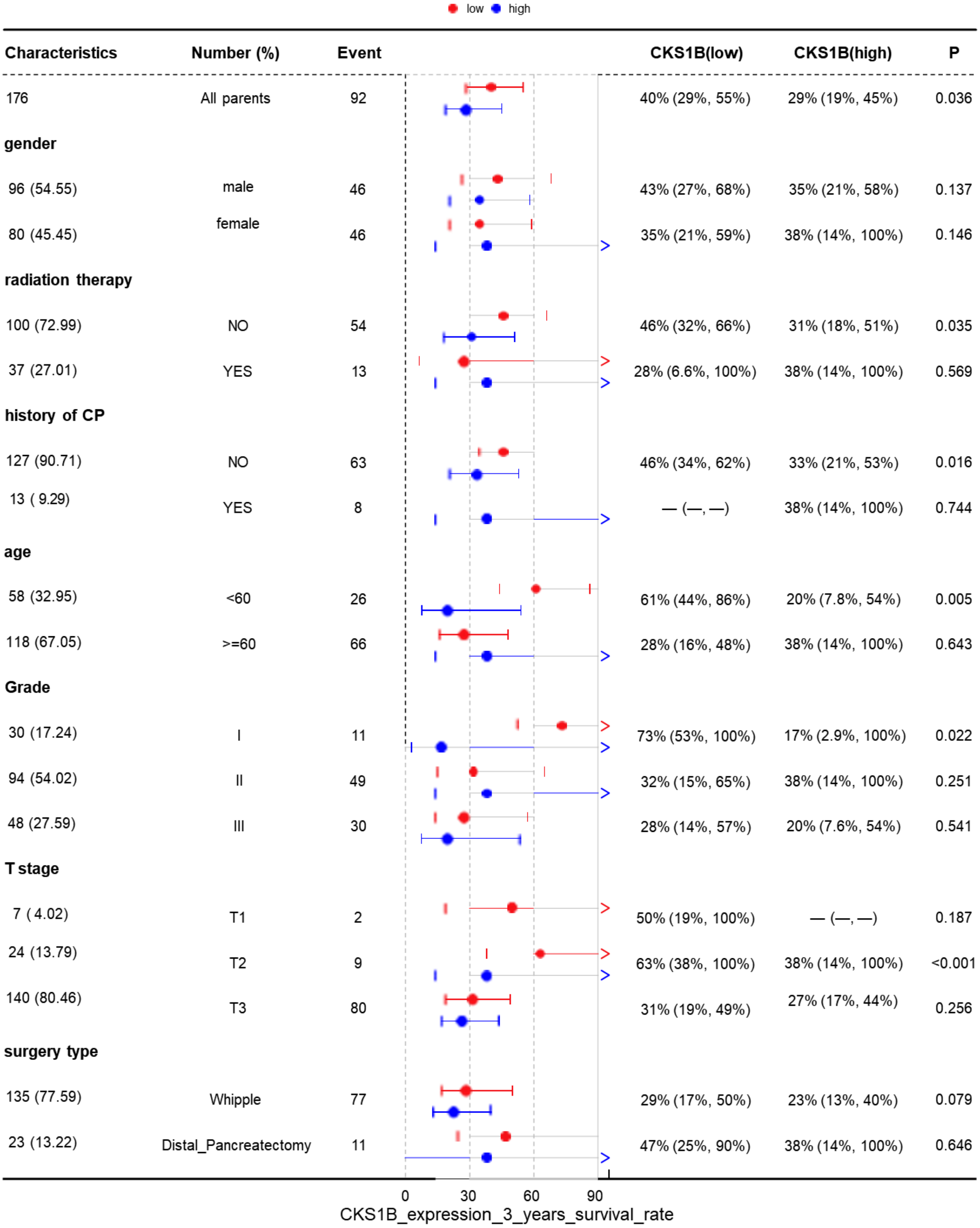

Characteristics	Number (%)	Event	CKS1B(low)	CKS1B(high)	P
176	All parents	92	40% (29%, 55%)	29% (19%, 45%)	0.036
**gender**					
96 (54.55)	male	46	43% (27%, 68%)	35% (21%, 58%)	0.137
80 (45.45)	female	46	35% (21%, 59%)	38% (14%, 100%)	0.146
**radiation therapy**					
100 (72.99)	NO	54	46% (32%, 66%)	31% (18%, 51%)	0.035
37 (27.01)	YES	13	28% (6.6%, 100%)	38% (14%, 100%)	0.569
**history of CP**					
127 (90.71)	NO	63	46% (34%, 62%)	33% (21%, 53%)	0.016
13 (9.29)	YES	8	— (—, —)	38% (14%, 100%)	0.744
**age**					
58 (32.95)	<60	26	61% (44%, 86%)	20% (7.8%, 54%)	0.005
118 (67.05)	>=60	66	28% (16%, 48%)	38% (14%, 100%)	0.643
**Grade**					
30 (17.24)	I	11	73% (53%, 100%)	17% (2.9%, 100%)	0.022
94 (54.02)	II	49	32% (15%, 65%)	38% (14%, 100%)	0.251
48 (27.59)	III	30	28% (14%, 57%)	20% (7.6%, 54%)	0.541
**T stage**					
7 (4.02)	T1	2	50% (19%, 100%)	— (—, —)	0.187
24 (13.79)	T2	9	63% (38%, 100%)	38% (14%, 100%)	<0.001
140 (80.46)	T3	80	31% (19%, 49%)	27% (17%, 44%)	0.256
**surgery type**					
135 (77.59)	Whipple	77	29% (17%, 50%)	23% (13%, 40%)	0.079
23 (13.22)	Distal_Pancreatectomy	11	47% (25%, 90%)	38% (14%, 100%)	0.646

Supplementary Figure 1

Survival analysis based on CKS1B expression in pancreatic cancer patients using data from the TCGA database. This forest plot illustrates the 3-year survival rates segmented by CKS1B expression levels (low vs. high) across various clinical characteristics: history of radiotherapy, chronic pancreatitis history, age (<60 years), early-stage PDAC classification (Grade I), and T2 stage. Statistically significant associations (P-values < 0.05) are noted for these groups. Red dots represent high CKS1B expression, while blue dots represent low expression.

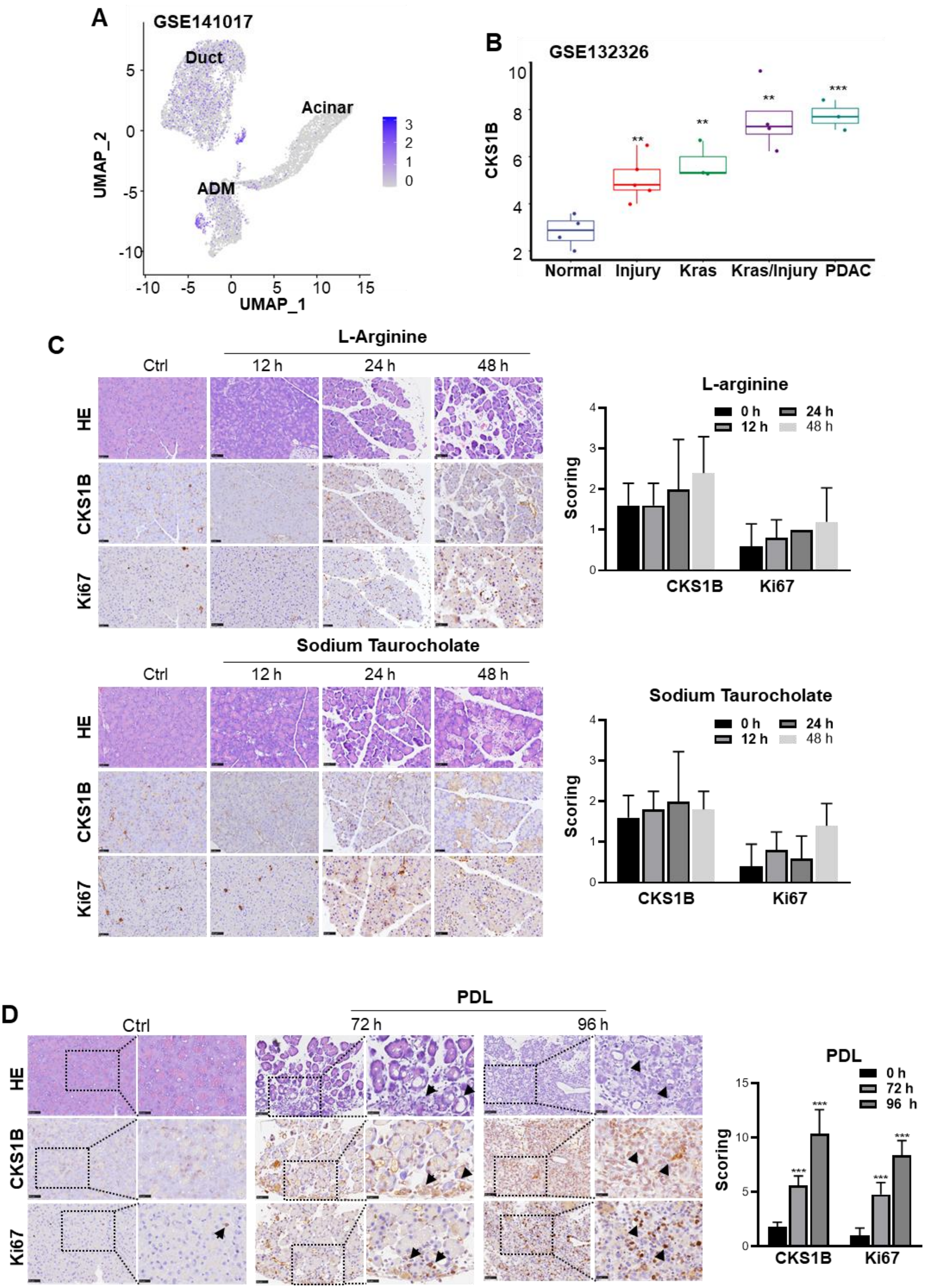
A
GSE141017
Duct
Acinar
ADM
UMAP_2
UMAP_1
B
GSE132326
CKS1B
Normal
Injury
Kras
Kras/Injury
PDAC
C
L-Arginine
Ctrl
12 h
24 h
48 h
HE
CKS1B
Ki67
L-arginine
0 h
12 h
24 h
48 h
Scoring
Sodium Taurocholate
D
PDL
72 h
96 h
0 h
72 h
96 h

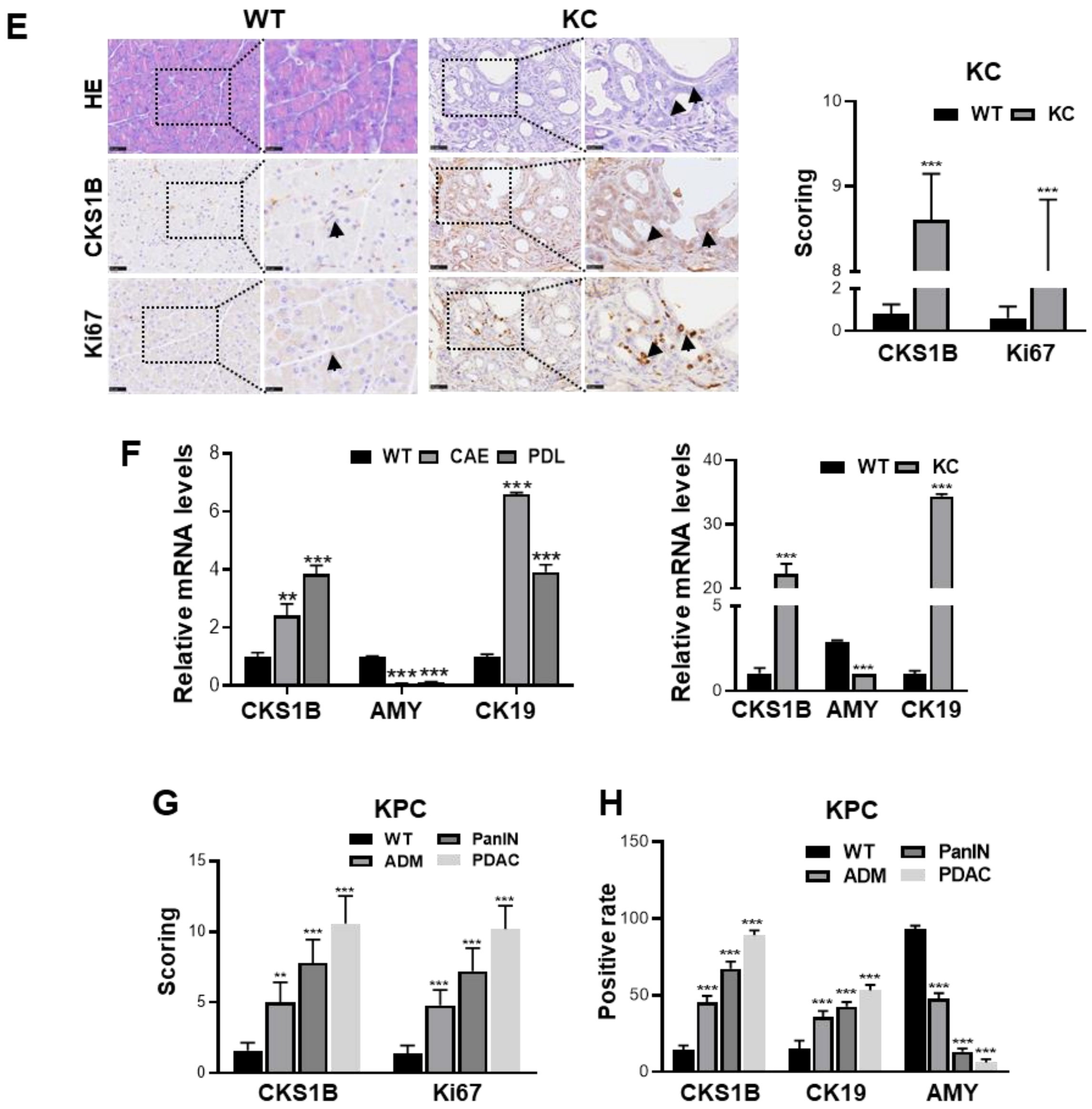
E
WT
KC
HE
CKS1B
Ki67
KC
WT KC
Scoring
10
9
8
2
0
***
***
CKS1B
Ki67
F
Relative mRNA levels
WT CAE PDL
8
6
4
2
0
**
***
***
***
***
***
CKS1B
AMY
CK19
Relative mRNA levels
WT KC
40
30
20
5
0
***
***
***
CKS1B
AMY
CK19
G
KPC
WT
PanIN
ADM
PDAC
Scoring
15
10
5
0
**
***
***
***
***
***
CKS1B
Ki67
H
KPC
WT
PanIN
ADM
PDAC
Positive rate
150
100
50
0
CKS1B
CK19
AMY

Supplementary Figure 2

Analysis of CKS1B Upregulation and its Correlation with Cell Proliferation in PDAC Development.

(**A**) Analysis of single-cell sequencing data from GSE141017 in mice demonstrates CKS1B upregulation in ADM lesions compared to normal acinar cells.

(**B**) RNA sequencing from GSE132326 tracks the progression from pancreatitis to neoplasm in mice, showing a gradual increase in CKS1B expression during PDAC development, particularly in the presence of Kras mutations, p=3.9e-05

(C) Construction of systemic inflammatory AP model (Left, L-ARG) and cholestatic AP model (Right, Sodium Taurocholate). The number of animals per group was 4, and the number of independent in vitro experiments was 3. Immunohistochemical analysis demonstrated that both CKS1B and the proliferation marker Ki67 remained unchanged in the AP lesions.

(**D**) Immunohistochemistry results indicated that ADM lesions occurred 72 and 96 hours after PDL surgery, with an upregulation of both CKS1B and Ki67 within these lesions compared to the control. These findings are consistent with those presented in Figure 2A.

(**E**) IHC demonstrated the upregulation of both CKS1B and Ki67 in ADM lesions of 30-week-old KC mice, a spontaneous ADM model, with WT mice serving as normal controls.

(**F**) Using WT mice as controls, RT-PCR analysis quantified the upregulation of CKS1B and CK19, and the downregulation of AMY, in pancreatic precancerous lesions from CAE, PDL, and KC mice.

(G) Quantitative IHC assays were used to assess the upregulation of CKS1B and Ki67 in 33-week-old KPC mice as pancreatic lesions progressed from ADM and PanIN to PDAC, with wild-type (WT) mice serving as normal controls.

(H) Quantification of multiplex immunohistochemistry was performed in 33-week-old KPC mice to assess the upregulation of CKS1B and CK19 as pancreatic lesions progressed from ADM and PanIN to PDAC, accompanied by a downregulation of amylase expression. Wild-type (WT) mice served as normal controls.

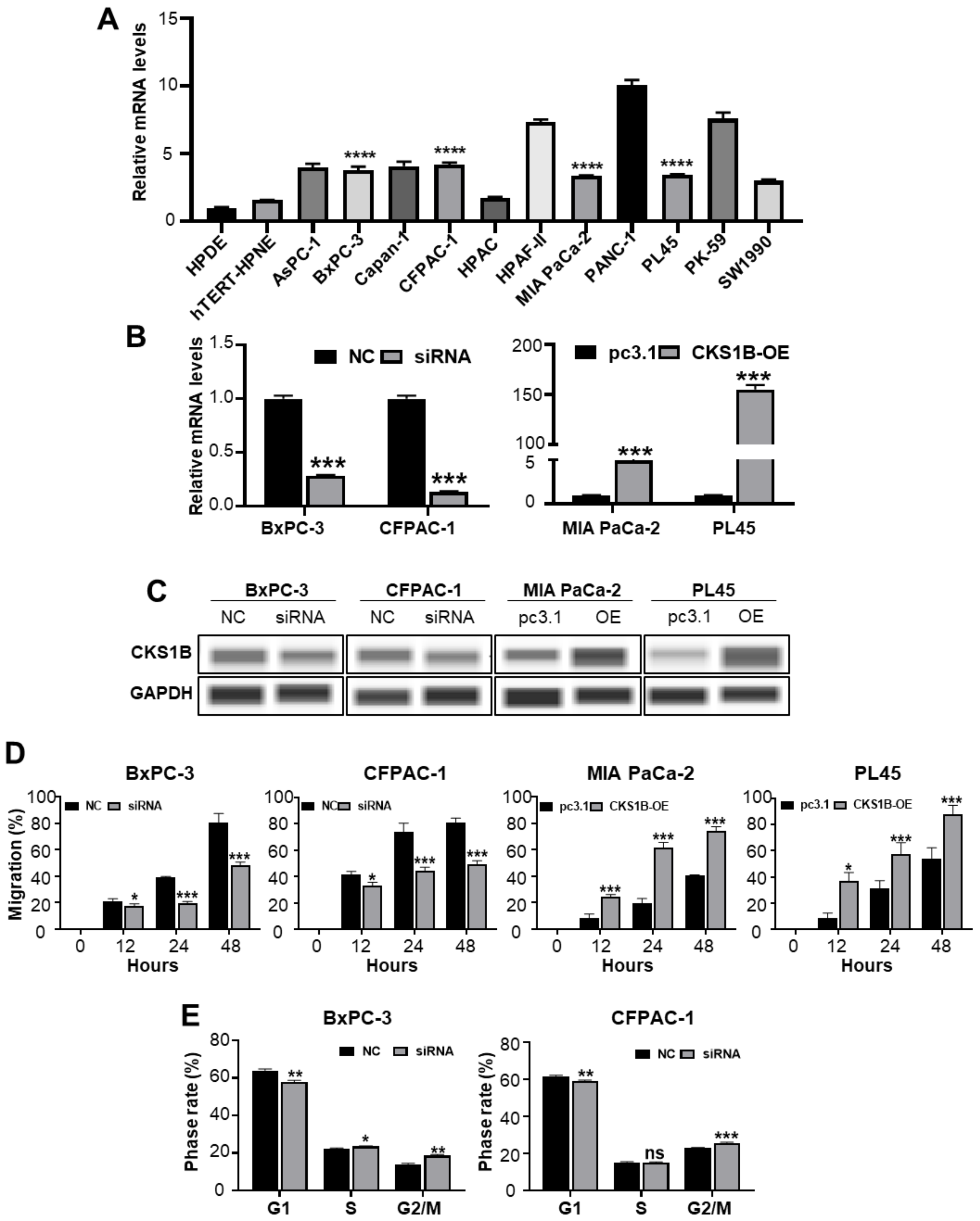
A
Relative mRNA levels
HPDE
hTERT-HPNE
AsPC-1
BxPC-3
Capan-1
CFPAC-1
HPAC
HPAF-II
MIA PaCa-2
PANC-1
PL45
PK-59
SW1990
B
NC
siRNA
pc3.1
CKS1B-OE
BxPC-3
CFPAC-1
MIA PaCa-2
PL45
C
BxPC-3
CFPAC-1
MIA PaCa-2
PL45
NC
siRNA
pc3.1
OE
CKS1B
GAPDH
D
BxPC-3
CFPAC-1
MIA PaCa-2
PL45
Migration (%)
Hours
E
BxPC-3
CFPAC-1
Phase rate (%)
G1
S
G2/M
ns

Supplementary Figure 3

CKS1B Overexpression Enhances Growth and Migration of PDAC Cells

(**A**) Confirmation of CKS1B mRNA expression in various PDAC cell lines. RT-PCR quantified CKS1B mRNA levels in 12 pancreatic cancer cell lines, with HPDE cells serving as normal controls. *$P < 0.05$, **$P < 0.01$, ***$P < 0.001$ vs. HPDE.

BxPC-3 and CFPAC-1 cells were transfected with CKS1B-siRNA, using NC as controls, while MIA PaCa-2 cells were transfected with a CKS1B overexpression vector, using pc3.1 as controls. CKS1B expression was quantified using (**B**) RT-PCR and (**C**) Western Blot analyses.

(**D**) Quantitative analysis of wound closure in each group demonstrated the impact of CKS1B on cell migration in the wound healing assay

(**E**) Quantitative analysis of cell cycle distribution in PDAC cells assessed the proportion of cells in each phase. Results indicate that CKS1B significantly hindered the transition from G2 to mitosis. Data are presented as mean ± SD from at least three independent experiments.

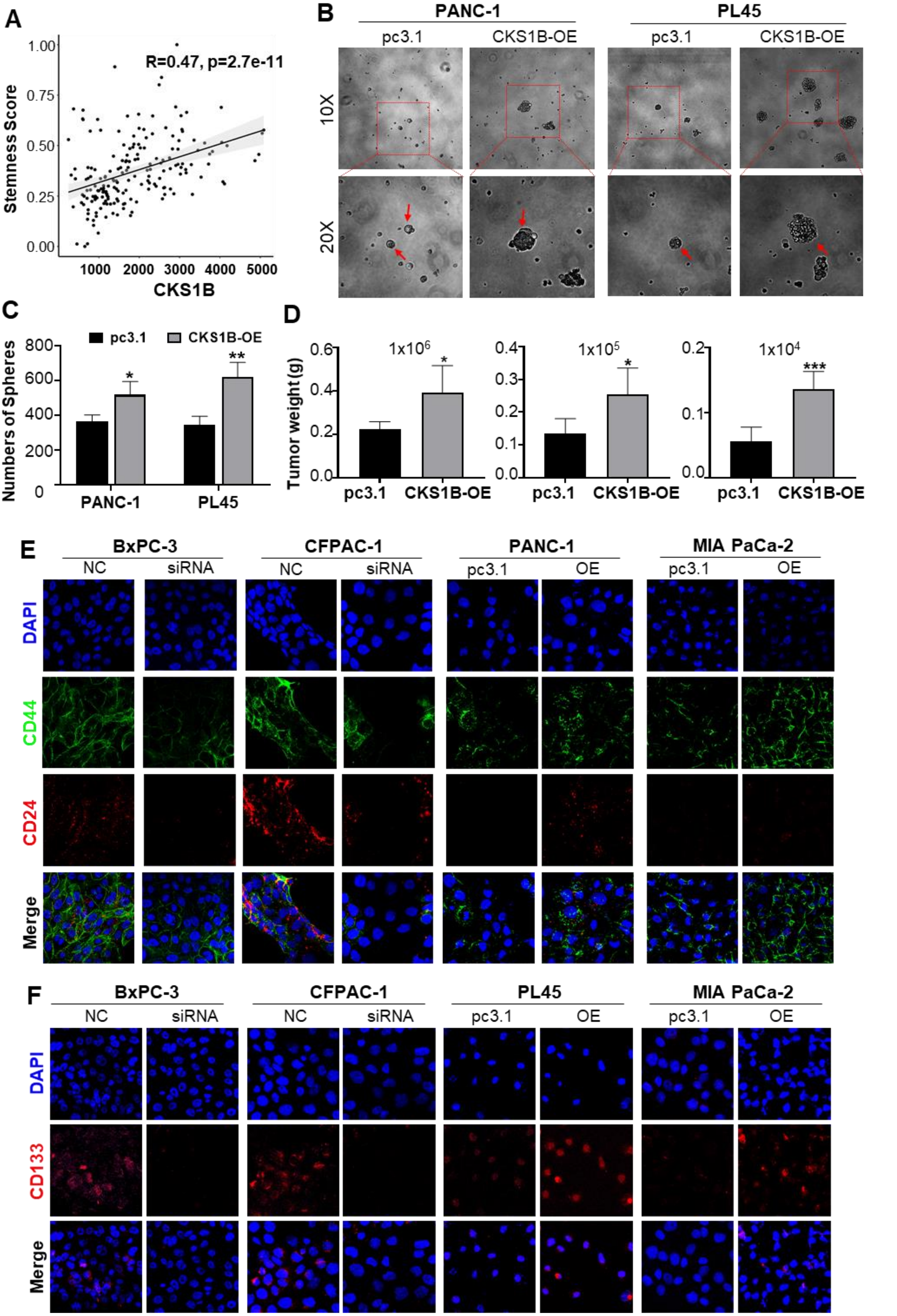
A
R=0.47, p=2.7e-11
Stemness Score
CKS1B
B
PANC-1
PL45
pc3.1
CKS1B-OE
10X
20X
C
pc3.1
CKS1B-OE
Numbers of Spheres
PANC-1
PL45
D
Tumor weight (g)
1x10^6
1x10^5
1x10^4
E
BxPC-3
CFPAC-1
PANC-1
MIA PaCa-2
NC
siRNA
pc3.1
OE
DAPI
CD44
CD24
Merge
F
BxPC-3
CFPAC-1
PL45
MIA PaCa-2
NC
siRNA
pc3.1
OE
DAPI
CD133
Merge

Supplementary Figure 4

CKS1B Expression Enhances the Stemness Properties of PDAC

(**A**) Analysis of RNA-sequencing data from TCGA demonstrates a correlation between CKS1B expression and stemness score in PDAC.

(**B**) The sphere formation assay demonstrated increased tumor sphere size and density in PDAC cells overexpressing CKS1B compared to vector control (pc3.1), indicating enhanced sphere-forming ability.

(**C**) *In vivo* limiting dilution assays on C57BL/6J mice injected with Panc02 cells overexpressing CKS1B or vector control (pc3.1) demonstrated that CKS1B overexpression increases the number of tumor-initiating cells, as evidenced by increased tumor weights.

(**D**) The proportion of $CD24^{+}CD44^{+}$ cells in CKS1B knockdown and CKS1B-overexpressing PDAC cells was assessed using cellular immunofluorescence. Results indicated that CKS1B expression affects the ratio of pancreatic cancer stem cells (PCSCs). Scale bars: 20 μm.

(**E**) Similarly, the proportion of $CD133^{+}$ cells in PDAC cell lines corroborated these results. Scale bars: 20 μm.

Zhang *et al*., Supplementary Figure 5

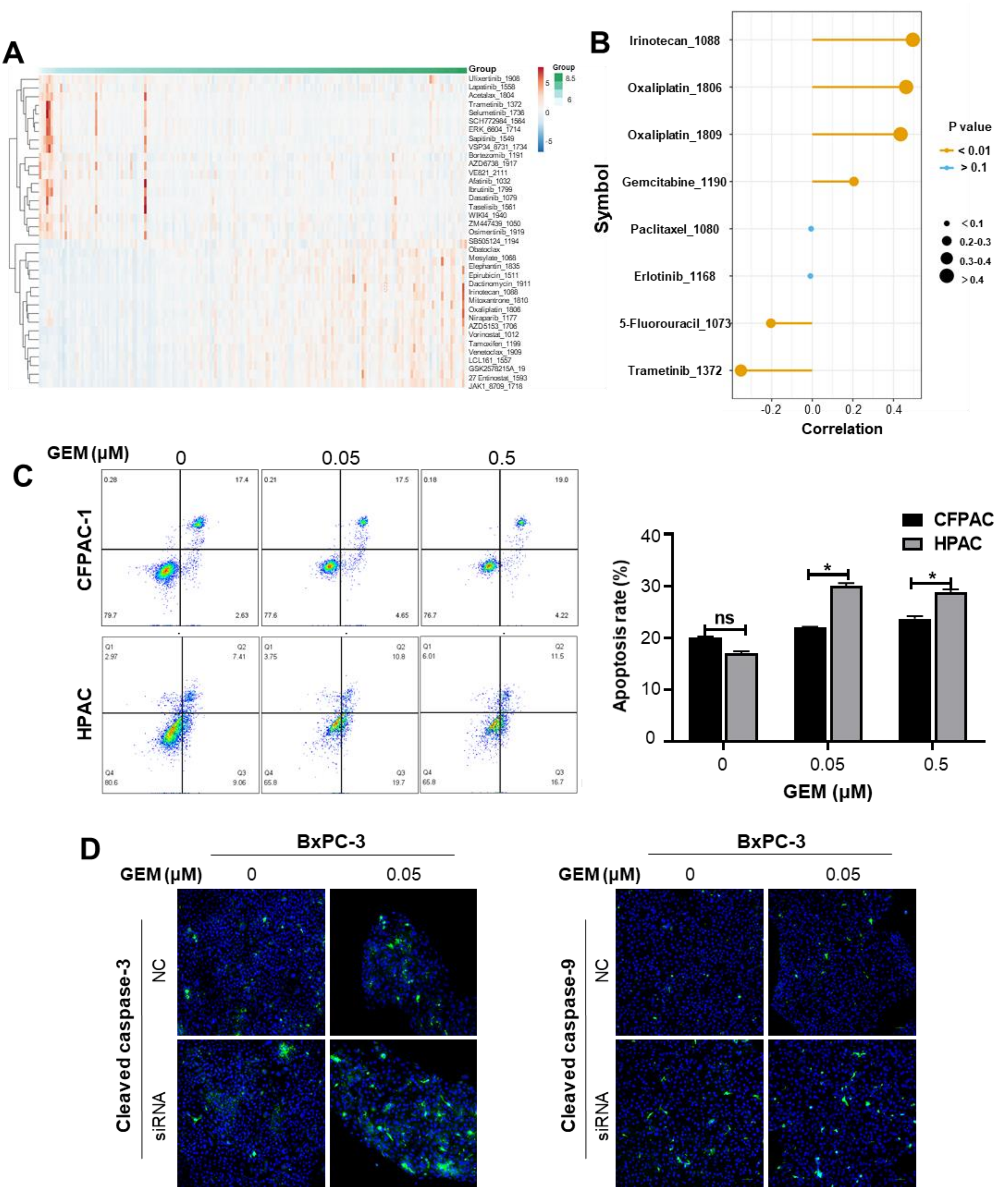

Supplementary Figure 5

CKS1B Mediates Drug Resistance in PDAC Cells by Blocking Gemcitabine and Oxaliplatin-Induced Apoptosis

(**A**) Heatmap plot exhibiting the top 20 drugs that are positively and negatively correlated with the expression of CKS1B. Predicted IC50 of selected drugs after being centered and scaled in the row direction for each sample is represented by color. Group showing the expression of CKS1B in samples from TCGA-PAAD cohort.

(**B**) Lollipop plot depicts correlations between CKS1B expression and eight chemotherapy drugs in pancreatic cancer. Dot size reflects Spearman's R value; color indicates significance: orange (#E69F00) for $P < 0.01$, , blue (#56B4E9) for $P > 0.1$.

(**C**) Flow cytometry analysis indicated that HAPC cells (low CKS1B expression) exhibited higher apoptosis rates compared to CFPAC-1 cells (high CKS1B expression) under varying concentrations of Gemcitabine, suggesting that CKS1B expression is closely associated with the sensitivity of PDAC cells to Gemcitabine.

(**D**) Immunofluorescence analysis revealed higher activation of cleaved caspase-3 and cleaved caspase-9 in CKS1B-knockdown cells compared to control (NC). This effect was most pronounced in cells co-treated with gemcitabine, further validating that CKS1B mediates drug resistance in PDAC cells by inhibiting Gemcitabine and Oxaliplatin-induced apoptosis. Scale bars: 100 μm.

Zhang *et al*., Supplementary Figure 6

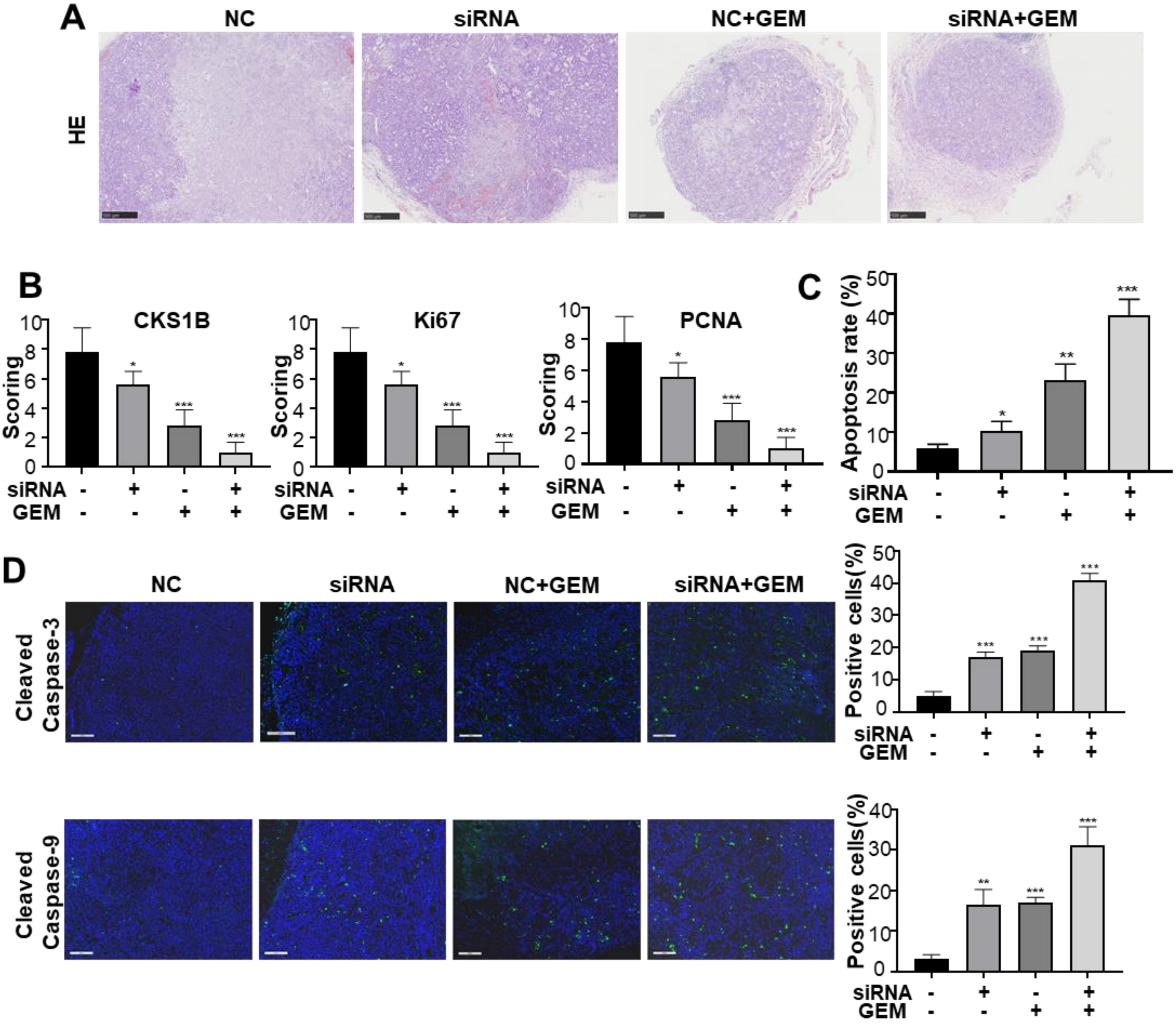

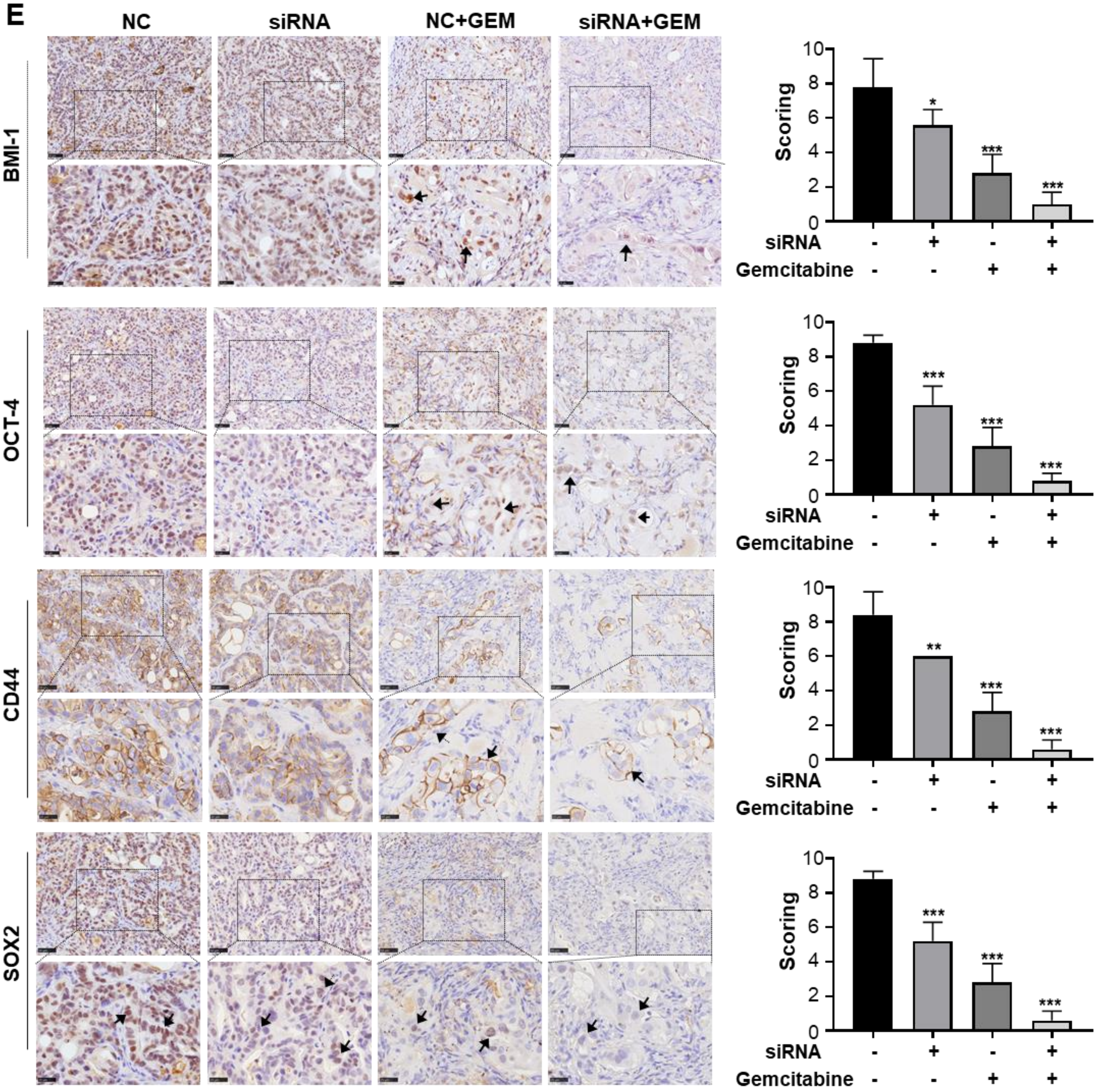
E
NC
siRNA
NC+GEM
siRNA+GEM
BMI-1
OCT-4
CD44
SOX2
Scoring
siRNA
Gemcitabine
*
**
***

Supplementary Figure 6

CKS1B Enhances Proliferation, Resistance, and Stemness of PDAC *In Vivo*

(**A**) Histological analysis on subcutaneous tumors revealed a smaller necrotic area in siRNA and siRNA +GEM groups compared with controls, indicating slower tumor growth. Scale bars: 500 μm.

(**B**) Pathology scoring of IHC staining in tumor tissues revealed that CKS1B knockdown led to decreased CKS1B expression and reduced proliferation markers (Ki67, PCNA). (**C**) Additionally, TUNEL assays quantified an increased apoptosis rate, (**D**) Immunofluorescence analysis showed increased activation of cleaved caspase-3 and cleaved caspase-9. Scale bars: 200 μm. These effects were further amplified upon co-treatment with gemcitabine, suggesting that CKS1B knockdown inhibits proliferation and enhances tumor sensitivity to gemcitabine *in vivo*.

(**E**) To further confirm that CKS1B modulates cancer stem cell (CSC) resistance to chemotherapy in PDAC, IHC analysis revealed decreased expression of PCSCs markers (BMI-1, OCT-4, CD44, and SOX2) in CKS1B-knockdown tumors. This downregulation was further pronounced following combination treatment with gemcitabine.

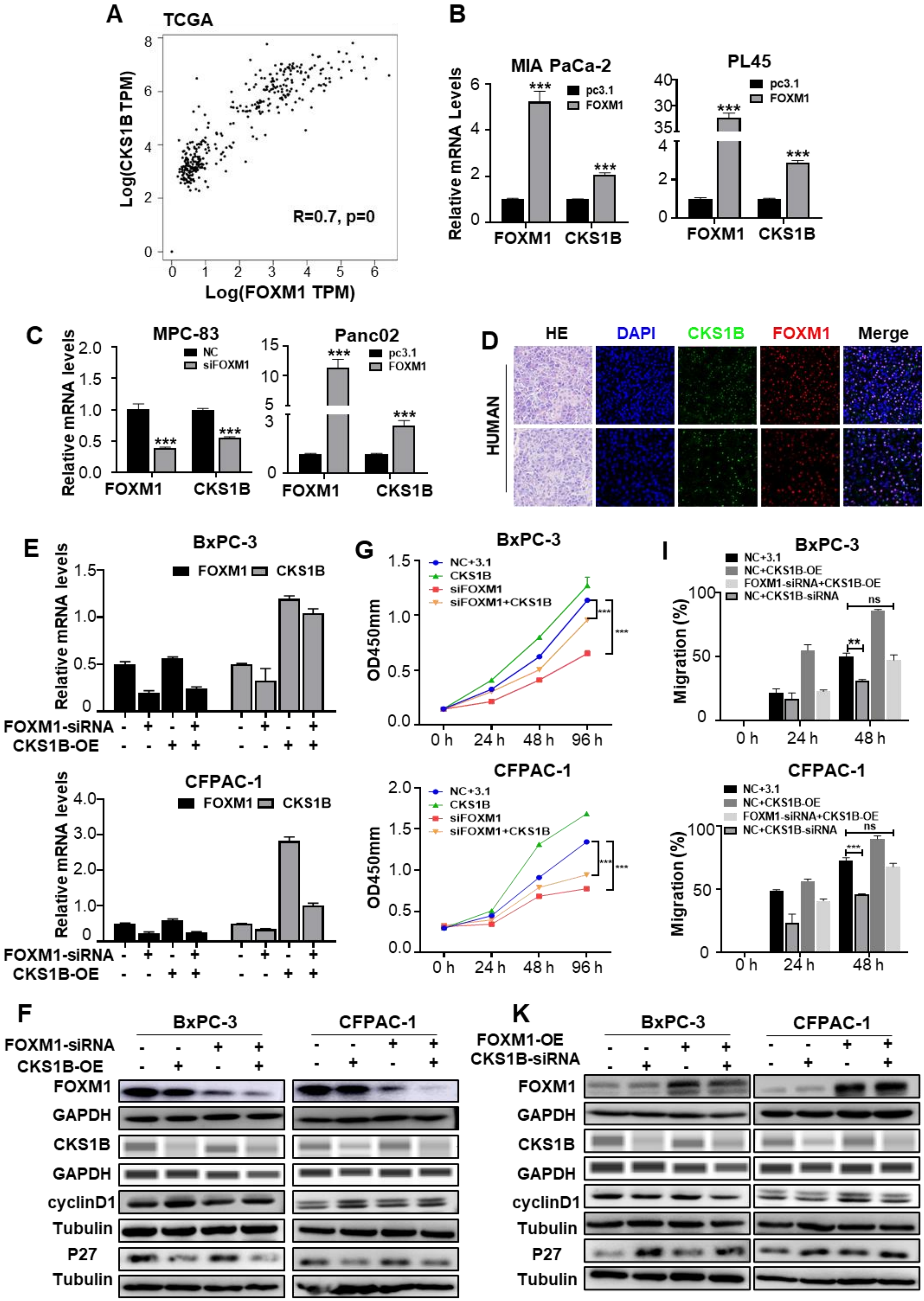
A
TCGA
Log(CKS1B TPM)
Log(FOXM1 TPM)
R=0.7, p=0
B
MIA PaCa-2
PL45
Relative mRNA Levels
pc3.1
FOXM1
FOXM1 CKS1B
C
MPC-83
Panc02
Relative mRNA levels
NC
siFOXM1
D
HE DAPI CKS1B FOXM1 Merge
HUMAN
E
BxPC-3
CFPAC-1
FOXM1-siRNA
CKS1B-OE
G
OD450mm
NC+3.1
CKS1B
siFOXM1
siFOXM1+CKS1B
0 h 24 h 48 h 96 h
I
Migration (%)
NC+3.1
NC+CKS1B-OE
FOXM1-siRNA+CKS1B-OE
NC+CKS1B-siRNA
ns
F
K
FOXM1-OE
CKS1B-siRNA
FOXM1
GAPDH
CKS1B
cyclinD1
Tubulin
P27

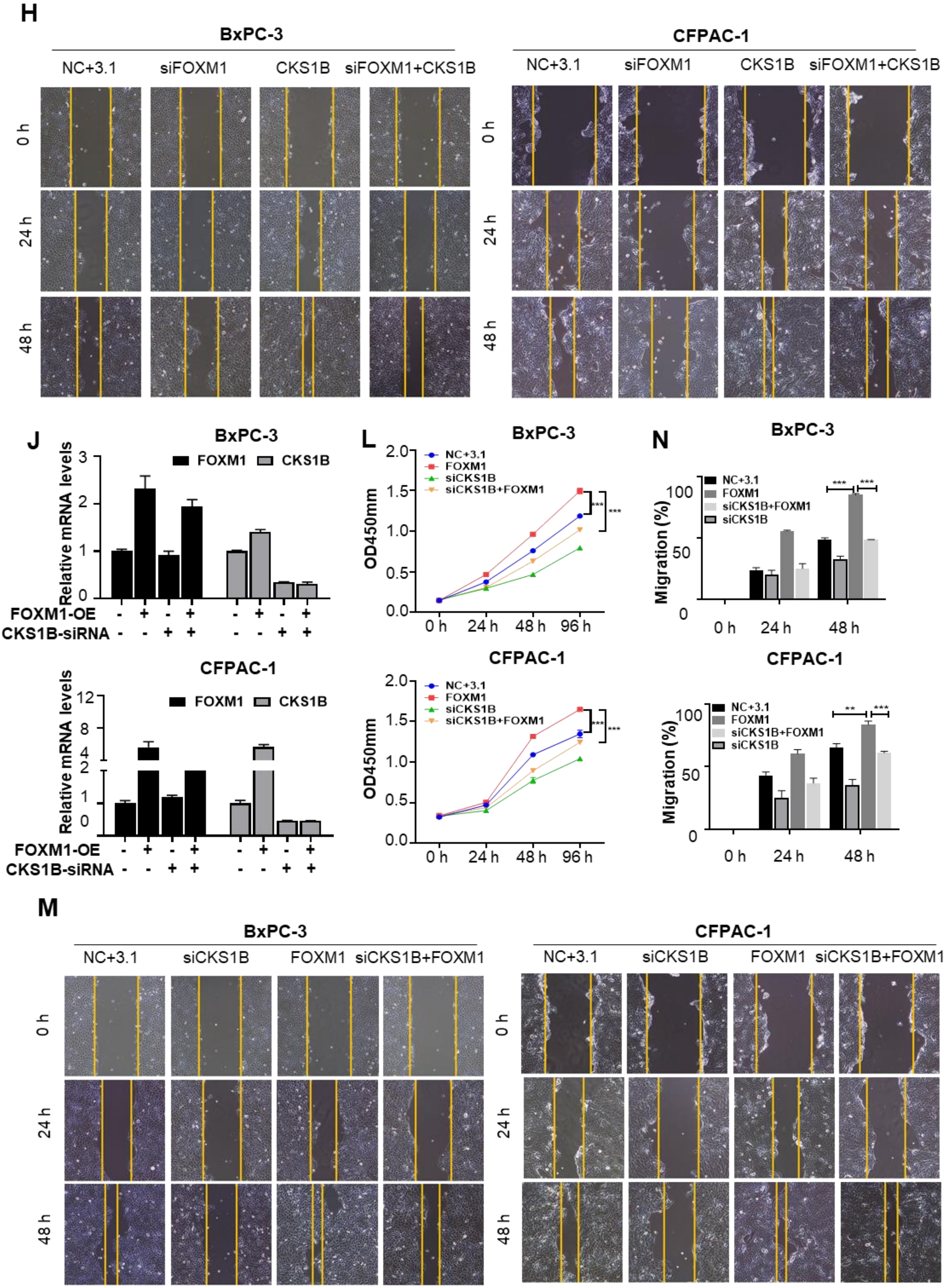
H
BxPC-3
CFPAC-1
NC+3.1
siFOXM1
CKS1B
siFOXM1+CKS1B
0 h
24 h
48 h
J
BxPC-3
CFPAC-1
FOXM1
CKS1B
Relative mRNA levels
FOXM1-OE
CKS1B-siRNA
L
OD450mm
NC+3.1
FOXM1
siCKS1B
siCKS1B+FOXM1
0 h
24 h
48 h
96 h
N
Migration (%)
M
siCKS1B
FOXM1
siCKS1B+FOXM1

Supplementary Figure 7

CKS1B Mediates FOXM1-Regulated Cell Proliferation, Migration and Stemness

(**A**) Analysis from TCGA database demonstrates a correlation between CKS1B expression and stemness score in PDAC.

(**B-C**) Consistent with the results shown in Fig. 7A, overexpression or knockdown of FOXM1 in human PDAC cells (PL45, MIA PaCa-2) or mouse PDAC cells (Panc02, MPC-83) resulted in corresponding changes in CKS1B expression

(**D**) Immunofluorescence co-localization of FOXM1 and CKS1B in human PDAC tissues revealed that both proteins are localized in the nucleus, suggesting a potential functional interaction. Scale bars: 50 μm

(**E-F**) In BxPC-3 and CFPAC-1 cells transfected with a CKS1B-overexpression vector and FOXM1-siRNA, with NC+pc3.1 serving as the control, RT-PCR and Western Blot analyses were used to assess the levels of CKS1B, FOXM1, and the cell cycle marker Cyclin D1and P27.

(**G**) CCK8 proliferation assays demonstrated that CKS1B-overexpression reversed the proliferation reduction caused by FOXM1-siRNA. (**H-I**)

Additionally, wound healing analysis confirmed and quantified that CKS1B-overexpression also reversed the migration decrease induced by FOXM1-siRNA. Scale bars: 400 μm.

(**J-K**) In BxPC-3 and CFPAC-1 cells transfected with a FOXM1-overexpression vector and CKS1B-siRNA, with NC+pc3.1 serving as the control, RT-PCR and Western Blot analyses were used to assess the levels of CKS1B, FOXM1, and the cell cycle marker Cyclin D1and P27.

(**L**) CCK8 proliferation assays showed that CKS1B knockdown reversed the increase in proliferation caused by FOXM1 overexpression. (**M-N**)

Additionally, wound healing analysis confirmed and quantified that CKS1B knockdown also reversed the enhanced migration induced by FOXM1 overexpression. Scale bars: 400 μm.

These results suggest that FOXM1 promotes a malignant phenotype characterized by enhanced proliferation and migration in PDAC through the mediation of CKS1B.
